# Habitat selection by Dall’s sheep is influenced by multiple factors including direct and indirect climate effects

**DOI:** 10.1371/journal.pone.0248763

**Published:** 2021-03-18

**Authors:** Jocelyn L. Aycrigg, Adam G. Wells, Edward. O. Garton, Buck Magipane, Glen E. Liston, Laura R. Prugh, Janet L. Rachlow

**Affiliations:** 1 Department of Fish and Wildlife Sciences, College of Natural Resources, University of Idaho, Moscow, Idaho, United States of America; 2 Lake Clark National Park and Preserve, National Park Service, Anchorage, Alaska, United States of America; 3 Cooperative Institute for Research in the Atmosphere, Colorado State University, Fort Collins, Colorado, United States of America; 4 College of the Environment, University of Washington, Seattle, Washington, United States of America; Wildlife Conservation Society Canada, CANADA

## Abstract

Arctic and boreal environments are changing rapidly, which could decouple behavioral and demographic traits of animals from the resource pulses that have shaped their evolution. Dall’s sheep (*Ovis dalli dalli*) in northwestern regions of the USA and Canada, survive long, severe winters and reproduce during summers with short growing seasons. We sought to understand the vulnerability of Dall’s sheep to a changing climate in Lake Clark National Park and Preserve, Alaska, USA. We developed ecological hypotheses about nutritional needs, security from predators, energetic costs of movement, and thermal shelter to describe habitat selection during winter, spring, and summer and evaluated habitat and climate variables that reflected these hypotheses. We used the synoptic model of animal space use to estimate parameters of habitat selection by individual females and calculated likelihoods for ecological hypotheses within seasonal models. Our results showed that seasonal habitat selection was influenced by multiple ecological requirements simultaneously. Across all seasons, sheep selected steep rugged areas near escape terrain for security from predators. During winter and spring, sheep selected habitats with increased forage and security, moderated thermal conditions, and lowered energetic costs of movement. During summer, nutritional needs and security influenced habitat selection. Climate directly influenced habitat selection during the spring lambing period when sheep selected areas with lower snow depths, less snow cover, and higher air temperatures. Indirectly, climate is linked to the expansion of shrub/scrub vegetation, which was significantly avoided in all seasons. Dall’s sheep balance resource selection to meet multiple needs across seasons and such behaviors are finely tuned to patterns of phenology and climate. Direct and indirect effects of a changing climate may reduce their ability to balance their needs and lead to continued population declines. However, several management approaches could promote resiliency of alpine habitats that support Dall’s sheep populations.

## Introduction

Species that live in extreme environments often exhibit traits that are tightly coupled with seasonal resource pulses. At high latitudes, reproduction across diverse taxa is synchronized with availability of forage and other resources necessary for offspring survival and growth [1]. Similarly, changes in space use and habitat selection coincide with seasonal shifts in climatic conditions and resource availability as individuals balance diverse resource needs and constraints across seasons [2, 3]. However, the behavioral and physiological specializations that facilitate adaptation to strongly seasonal environments also might render species less able to respond to changing conditions [1, 4, 5].

High latitude arctic and boreal environments are changing rapidly with the potential to decouple behavioral and demographic traits of animals from the resource pulses that have shaped their evolution. Temperatures in the Arctic and subarctic (i.e., above 60⁰ N latitude) are increasing at twice the rate of global means [6], while snow cover extent has decreased by about 10% since the late 1960s [7]. Snow-free periods are 5–6 days longer per decade [4, 8], and precipitation has increased about 1% per decade over the past 100 years [7], which influences not only depth, extent and persistence of snow cover [4], but also snow density and occurrence of rain-on-snow events [9, 10]. Climate change also can indirectly affect wildlife populations at high latitudes by altering the composition of ecological communities [11], including expansion of shrubs into tundra and alpine habitats [12, 13] and changes in plant phenology in arctic communities [14, 15]. Understanding of the effects of climate change on arctic-adapted wildlife species via both direct and indirect pathways is advancing.

Dall’s sheep (*Ovis dalli dalli*), which occur only in Alaska, USA, and the Yukon, Northwest Territories and British Columbia, Canada, are well adapted to arctic and subarctic alpine ecosystems; they can survive long, severe winters and reproduce during summers with short growing seasons [16–18]. In most populations, both sexes migrate seasonally between summer and winter ranges, and during spring, female Dall’s and Stone’s (*O*. *d*. *stonei*) sheep move to traditional lambing areas where they trade off forage availability for security from predators and select areas with shelter from harsh weather [18–22]. These ungulates are prey for multiple predators [23–25] and are an important harvested species for both subsistence and recreational hunters [26, 27]. However, some populations have declined markedly (i.e., 50–80%), raising concern about the influence of climate change on their demography and their ability to adapt to on-going and future changes in climate and distribution of resources [28–31].

Recent work has suggested that climate can exert a strong influence on the behavior and demography of Dall’s sheep, but many questions remain. Across Alaska, a decline in recruitment of lambs and survival of adults is strongly affected by spring weather, and the magnitude of the effect varies across latitudes [32–34]. In the Northern Richardson Mountains of the Yukon in Canada, multiple factors such as potential competition for resources with other herbivores, predation, and emerging diseases could be contributing to observed population declines, however, the effects of documented increases in temperature and precipitation related to climate change remain unknown [30]. Nonetheless, snow properties during winter have been shown to influence movement, space use, energetic costs, and forage accessibility by Dall’s sheep across multiple scales, which could reduce adult survival and population persistence [35]. Severe weather events, such as icing and snow cover persisting into late spring, have reduced the amount of available habitat during critical periods potentially leading to declines in sheep populations across Alaska, including in Gates of the Arctic, Denali, and Lake Clark National Parks and Preserves [28, 32, 36]. Although previous studies have characterized seasonal patterns of habitat selection by Dall’s sheep [20, 25, 37, 38], only Mahoney et al. [35] and Cosgrove et al. [39] incorporated climate variables on a daily basis. Consequently, an understanding of how current climatic conditions influence real-time habitat selection by Dall’s sheep throughout the year is lacking, and such information is critical for evaluating how changes in climate over time might affect availability and quality of selected habitats.

**Fig 1:**
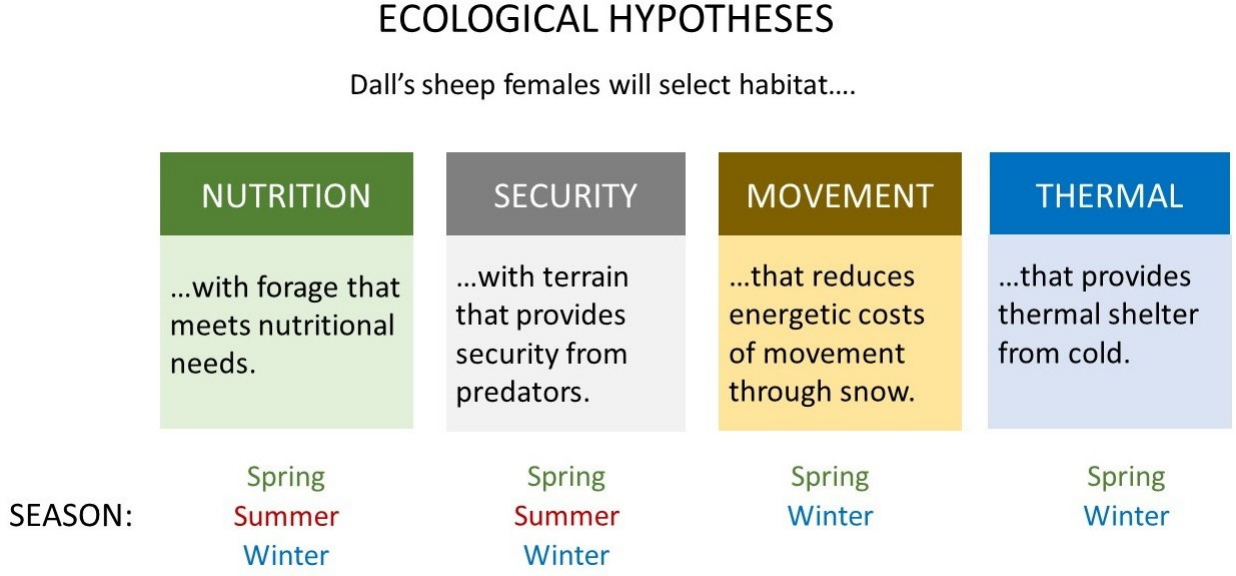
Ecological hypotheses to explain habitat selection across seasons by Dall’s sheep females (*Ovis dalli dalli*) at Lake Clark National Park and Preserve, Alaska, USA.

Our goal was to investigate how both ecological and climate variables shape habitat selection by Dall’s sheep on a fine temporal scale (i.e., 7-hr intervals) across the annual cycle, thereby providing a foundation for assessing vulnerability to changing climatic conditions. To achieve this goal, we developed ecological hypotheses about nutritional needs, security from predators, energetic costs of movement, and thermal shelter to describe habitat selection during winter, spring, and summer to provide context for assessing how climate variables influence habitat selection (Fig 1). Our work is unique because we used a quantitative modeling approach to test ecological hypotheses about habitat selection in addition to evaluating individual habitat variables. Our ecological hypotheses were composed of habitat and climate variables that have been shown to be important to the fitness of Dall’s sheep across seasons [18, 19, 22, 25, 37, 38, 40, 41]. Furthermore, we used a spatially distributed snow-evolution modeling system, which estimated climate variables, including snow depth, snow extent, wind speed, and air temperature (i.e., SnowModel) [42, 43] at daily intervals. We expected that climate variables would influence daily habitat selection strongly and directly during the spring and winter seasons and less so during summer. Our modeling approach allowed us to assess this in the context of our ecological hypotheses of habitat selection by Dall’s sheep.

We used GPS locations of adult Dall’s sheep collected during 2005–2008 in Lake Clark National Park and Preserve, Alaska, and synoptic modeling, to evaluate how females selected resources across seasons to meet their requirements for nutrition, security, thermal shelter, and energy. We hypothesized that during the spring lambing season, females would select for rugged terrain associated with security and for less extreme thermal conditions (higher ambient temperatures and lower windspeeds) to provide thermal shelter for neonates. Dall’s sheep are relatively short-limbed and moving through snow increases energy expenditure of locomotion [44], and indeed both empirical studies [10] and modeled movement patterns [35] demonstrated that snow properties affect movement of Dall’s sheep. Therefore, we expected that females would avoid areas with snow cover and select for shallower snow depths during both the winter and spring seasons to reduce the energetic costs of movement. We hypothesized that nutritional needs and forage availability would influence habitat selection during summer when energetic requirements are elevated due to lactation and the need for females to recuperate body condition during the short growing season. Additionally, because lambs likely remain vulnerable to predators throughout the summer, we hypothesized that security also would influence habitat selection. We developed models incorporating both habitat and climate variables to test these ecological hypotheses (Fig 1; Table 1) and provide a foundation for understanding and anticipating how environmental change might influence the abundance and distribution of seasonally suitable habitat for Dall’s sheep populations under future climates.

**Table 1:** Ecological hypotheses, expectations, and associated habitat and climate variables (grouped by categories: vegetative, topographic, and climate) used for analyses of seasonal habitat selection by female Dall’s sheep (*Ovis dalli dalli*) at Lake Clark National Park and Preserve, Alaska, USA. From these ecological hypotheses and habitat variables, we created a set of models for each season using single and multiple ecological hypotheses and model selection procedures to identify habitat variables to retain for each season (see S2 Text for details). See S1 Table for a description of each habitat and climate variable.

Ecological hypotheses	Expectation	Season	Vegetative	Topographic	Climate
Nutrition	Females will select areas with available forage, less snow and shallower snow depths to meet their nutritional needs.	Spring	alpine dwarf scrub^a^		snow depth (Winter) snow extent^a^ (Spring)
		Summer	shrub/scrub^a^		
		Winter	NDVI^b^		
Security	Females will select terrain features including steep slopes and rugged terrain that facilitates detection of and escape from predators.	Spring Summer Winter		elevation	
				slope	
				ruggedness^c^	
				distance to escape terrain mean slope x ruggedness^d^	
Movement	Females will select slopes with less snow cover and shallower depths to reduce energetic costs of movement.	Spring Winter		sine aspect^e^ (Winter)	snow depth snow extent^a^ (Spring)
Thermal	Females will select areas with warmer temperatures, greater solar radiation, lower wind speeds, and less snow cover to reduce energetic costs of thermoregulation.	Spring Winter		sine aspect^e^	air temperature
					snow depth
					snow extent^a^ (Spring)
					solar radiation wind speed

^a^percent area evaluated within 270-m radius circular buffer

^b^normalized difference vegetation index

^c^evaluated across 7x7 pixels at 30-m resolution and based on Sappington et al. [45]

^d^evaluated across 3x3 pixels at 30-m resolution, this is an interaction variable

^e^an index of eastness

## Methods

### Study area

**Fig 2:**
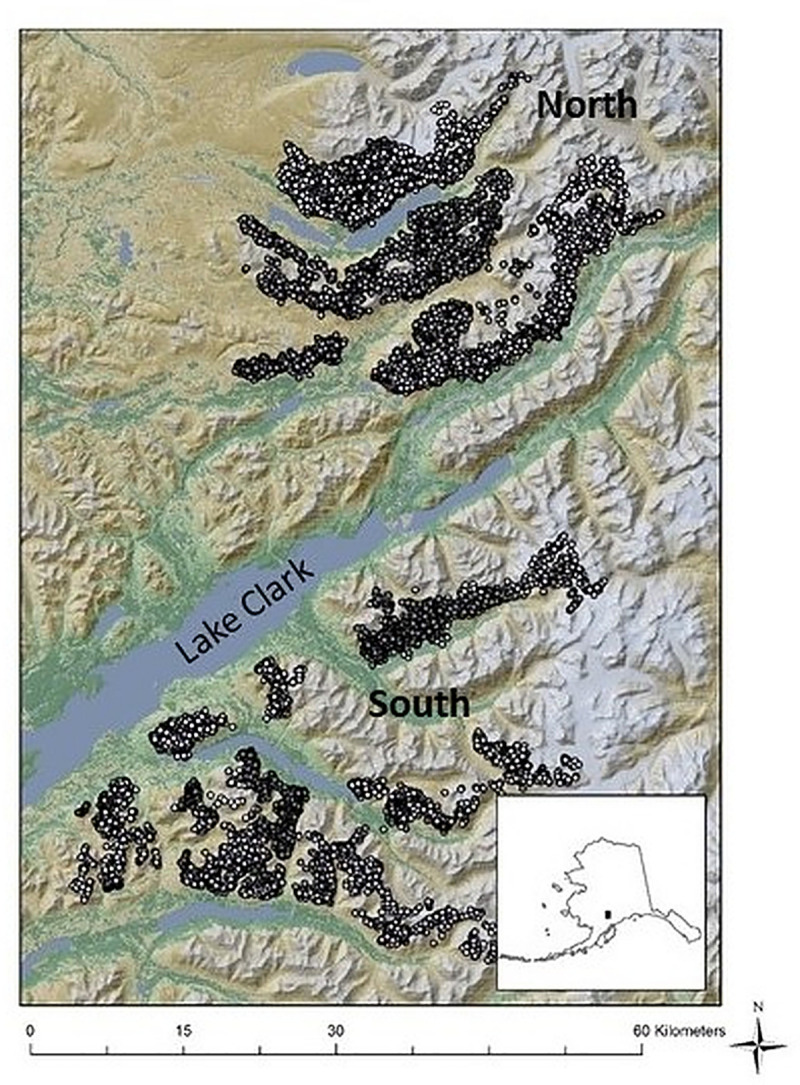
Study area of Lake Clark National Park and Preserve, Alaska, USA, with representative GPS points from adult female Dall’s Sheep (*Ovis dalli dalli*). Animals from two subpopulations were collared, one on the north side of Lake Clark (North region) and a separate group on the south side (South region).

Lake Clark National Park and Preserve (LCNPP) located at about 60⁰N and 154⁰W encompasses 1.6 million ha in southcentral Alaska, USA, and is situated on the southwestern edge of Dall’s sheep range. Although below the Arctic Circle, this region falls within the arctic and subarctic regions where climate is rapidly changing [6, 46]. Within LCNPP, Dall’s sheep inhabit both the Neacola Mountains in the Alaska Range northeast of Lake Clark and the Chigmit Mountains in the Aleutian Range on the southern side of Lake Clark (Fig 2). The mountainous terrain is rugged with elevations ranging from sea level to >3000 meters. About 44% of LCNPP is covered by snow, glacial ice, or sparsely vegetated gravel and bedrock with about 19% shrubland, 15% tundra, and 11% forest vegetation. The remaining 11% consists of grasslands, freshwater rivers and lakes, salt marshes, sedge meadows, and bogs (www.nsp.gov/lacl). During the time period for our study (2005–2008), seasonal values for mean temperature and mean total precipitation in the interior portion of LCNPP where Dall’s sheep occur were 6.8 to 8.9⁰ C and 13.5 to 33.9 mm during spring (May-June), 12.7 to 15.7⁰ C and 60.0 to 96.4 mm during summer (July–August), and -16.1 to -10.0⁰ C and 8.5 to 18.4 mm during winter (January–February [47]).

### Telemetry data

We evaluated movements and habitat selection by 20 adult female Dall’s sheep fitted with GPS telemetry collars in LCNPP, Alaska. The collared females occurred in two distinct regions of the Park (i.e., North and South of Lake Clark; Fig 2). All field data collected on Dall’s sheep were collected prior to the start of our research project. Our research project was not involved in the field data collection. Telemetry data were collected by National Park Service personnel from October 2005 to May 2008. Collars were programmed to record locations every 5–7 hours, and we used location data during the two full years when data were available (2006 and 2007). Mahoney et al. [35] used the same data set, however, we included data from spring and summer as well as winter. Like Mahoney et al. [35] we restricted our analysis to position fixes with positional dilution of precision (PDOP) values < 12.0 to eliminate locations with potentially large errors [48].

We used movement data to assess seasonal patterns of space use and delineate time periods reflecting different resource needs. We discerned 2 seasonal shifts in space use across the annual cycle that were consistent between years (S1 Fig). We chose dates for summer (1 July– 31 August) and winter (1 January– 28 February) during the middle of each of these seasons to avoid periods when sheep were in transition from summer to winter ranges (S1 Fig). To determine dates for the spring season when reproduction occurred, we relied on previous research for onset and duration of lambing [18, 49]; we chose 15 May– 24 June, which included the majority of births, to reflect a period of resource needs associated with reproduction.

### Spatial data

We developed GIS data layers for assessment of habitat selection that encompassed LCNPP at 90-m resolution. We started with 40 habitat data layers grouped into 3 categories: vegetative (*n* = 22), topographic (*n* = 11), and climate (*n* = 7; S1 Table). We derived vegetative data, which included the landcover types of ice, barren, deciduous, evergreen, mixed forest, alpine dwarf scrub, and scrub/shrub from the National Land Cover Database for Alaska (NLCD) [50]. We treated land cover as a categorical variable and estimated percent cover of each land cover type within circular buffers with radii of 135 m and 270 m centered around each pixel to quantify vegetation cover at multiple scales. We retained only alpine dwarf scrub and shrub/scrub for model development because use of other land cover types by Dall’s sheep was minimal. Alpine dwarf scrub often occurs at higher elevations in alpine areas with grasses, sedges, herbs, and non-vascular vegetation and describes areas dominated by shrubs <20 cm tall with shrub canopy >20% of total vegetation [50]. Shrub/scrub includes shrubs, young trees or trees stunted from environmental conditions and describes areas dominated by shrubs <5 m tall with shrub canopy >20% of total vegetation [50]. To assess temporal variation in plant phenology, we obtained estimates of the maximum value of the normalized difference vegetation index (NDVI) captured weekly at a 250-m resolution by USGS EROS eMODIS [51]. The NDVI estimates were available for each season (i.e., winter, spring, and summer) and have been filtered to ensure only valid NDVI values were included [51, 52].

We included elevation as a topographic variable and also derived base topographic variables from the 1-arc second National Elevation Dataset (NED) [53] resampled to 90 m (used the Project Tool/Environmental settings in ArcGIS 10.4, ESRI, Redlands, California). Derived variables included sine of aspect (an index of eastness), cosine of aspect (an index of northness), and distance to escape terrain, classified as slopes >30 degrees. We created an index of terrain ruggedness following Sappington et al. [45] at 3 resolutions (3 x 3, 5 x 5, and 7 x 7 pixels with 30-m pixels). We multiplied slope by the ruggedness index and calculated means and standard deviations for each of the 3 resolutions to represent complex, broken terrain often associated with security for mountain ungulates [17, 54, 55]. Finally, we also included 3 interaction terms, which described the interaction between barren areas based on NLCD [50] and distance to escape terrain and the interaction between mean slope and ruggedness (S1 Table). Our intent was to identify steep rocky areas used by females to provide security for lambs and evade predators [19, 56].

To evaluate the impact of climate on habitat selection by Dall’s sheep, we used SnowModel, a spatially distributed snow-evolution modeling system (S1 Text) [35, 42, 43]. SnowModel simulations were conducted using a daily time step during 1 September 2005–31 August 2008 over the spatial domain that included LCNPP, which produced daily estimates at 90-m resolution for snow depth, air temperature, and wind speed. SnowModel inputs were land cover from NLCD and topographic information from NED [50, 53]. We cross-referenced daily snow depth metrics with SNOTEL field station data from Telaquana Lake and Port Alsworth, in LCNPP, to validate results and calibrate SnowModel [42]. From the snow depth distributions, we calculated values of snow extent at multiple scales. We obtained a binary classification of whether snow was present within 90-m pixels and also estimated the percent of snow cover within 135 m and 270 m buffers. SnowModel outputs are based on the physical understanding of snow-evolution processes and features, and their interactions with the atmosphere and surrounding land surface (S1 Text) [35, 42].

Finally, because absorption of solar radiation differs between light surfaces (i.e., snow) and dark surfaces (i.e., bare ground), which influences snow extent, we estimated a daily solar radiation index (SRI) [57–61] for each day during 2006–2008. We used a spatial resolution of 90 m, which matched the output from SnowModel, and we obtained 365 (or 366 for leap years) raster layers indicating the average daily value of solar radiation. Our climate variables therefore consisted of snow depth, snow extent, percent snow extent within 135 meters and 270 meters, air temperature, wind speed, and SRI.

### Habitat selection analyses

To evaluate how Dall’s sheep females selected resources across seasons to meet their requirements for nutrition, security, thermal shelter, and energy, we first created ecological hypotheses that represented resource requirements for females during each season (Fig 1; Table 1), and we evaluated habitat variables that reflected those resource needs during each season. Preliminary data processing included evaluation of the univariate strength of predictor variables (some across multiple spatial scales), followed by tests for collinearity and removal of variables that were highly correlated (r ≥ 0.7). Using uncorrelated variables, we ran a synoptic model for each individual and each combination of predictor habitat variables for each Ecological Hypothesis x Season combination (n = 10; Table 1; see details in S2 Text). Our final step was to compare predictive ability across the seasonal model sets to identify the models that best explained selection within each season (spring, winter, and summer). Because these models were generated from *a priori* ecological hypotheses, we interpret the influence of climate variables on habitat selection in a broader context that encompasses ecological requirements for nutrition, security, thermal shelter, and energy.

We used the synoptic model of animal space use to estimate parameters of habitat selection by individual females and calculated likelihoods for the ecological hypotheses within each seasonal model set (Table 1) [62]. We chose to use the synoptic model because it allowed us to quantify both space use and habitat selection simultaneously across a season, while also incorporating daily time steps to represent more realistic effects of climate variables on habitat use. The synoptic modeling approach starts with a null model of space use that represents how individuals would use their habitat in absence of any effects from habitat or climate variables and quantifies any proportional change in the probability of use of an area attributable to the predictor variables (in our models, both habitat and climate variables). We used a bivariate normal distribution for the null model because our GPS locations indicated that individual female movements were generally within a central place during each season (S1 Fig) [62, 63]. Additionally, we chose the synoptic model, in part, because we could develop models of habitat selection by treating individuals as replicates to evaluate the population-level effects of each habitat or climate variable rather than conducting analyses solely at the population level by treating individual locations as replicates. We can also evaluate the strength of evidence for our *a priori* ecological hypotheses to further understand the ecological processes that influence seasonal habitat selection by Dall’s sheep. The synoptic model is unique because it estimates the home range size, home range shape, and the habitat selection coefficients as well as the interrelations with other individuals simultaneously by treating individual locations as replicates within a probability density function [62].

The synoptic model estimates the probability of finding an individual at a specific location as a function of multiple, spatially explicit habitat and climate variables within the area used by the individual. More specifically, the synoptic model estimates the probability of use of a particular location (f_u_(*x*)) for each individual from the product of availability of habitat and climate variables (a vector of both continuous and categorical variables) at that location (f_a_(*x*)) weighted by a resource selection function (*w(x)*) divided by a normalizing constant (*K*) where the weighting function (*w(x)* = exp(*b*_*0*_+*b*_*1*_*x*_*1*_+…+*b*_*p*_*x*_*p*_) is estimated by maximum likelihood, and the normalizing constant represents the sum of the weighting function across the entire landscape [62, 64]. This process converts the weighting function to the form of a probability distribution.

Models were developed that incorporated combinations of our spatial data that described the LCNPP environment (i.e., habitat and climate variables) and reflected our ecological hypotheses. These models were parameterized by maximum likelihood using the telemetry data collected from each individual at discrete times and our spatially explicit habitat and climate variables. Model output included values of Akaike’s information criterion corrected for small sample sizes (AIC_c_) for each model and each individual. To evaluate population-level effects, we used an information theoretic approach to select the model with the most support from the data by summing all AIC_c_ values for each model across individuals (detailed in S2 Text) [62]. Our availability landscape encompassed all of LCNPP, sampled at a 90-m resolution. Because GPS data were collected in two regions on the north and south side of Lake Clark (Fig 2), and it is possible that sheep might select habitat differently between these areas, we evaluated selection by individuals for the northern and southern regions separately. Sample sizes did not permit evaluating models separately by region and year. Since the synoptic model estimates habitat selection by individual sheep, we treated each individual as one observation from the population of sheep and used a Student’s t-test to determine if parameter estimates for each individual differed significantly from zero [65].

## Results

We tested ecological hypotheses of habitat selection by Dall’s sheep in two regions of LCNPP (north and south of the Lake; Fig 2) during winter, spring, and summer. We used locations from 20 female sheep fitted with GPS collars during 2006 (*n* = 18,096 locations) and 2007 (*n* = 19,927 location). After filtering the data to selected seasons and eliminating locations with potentially large errors, we retained 4,721 locations for modeling habitat selection during spring, 6,972 for summer, and 2,858 for winter (winter data were available only during 2007). Fifteen females provided two years of spring and summer data.

### Seasonal habitat selection

Dall’s sheep females selected habitat in response to multiple ecological requirements during all seasons. After eliminating correlated variables, we retained 14 habitat and climate variables (3 vegetative, 5 topographic, 5 climate, and 1 interaction) for use in the synoptic models to test our ecological hypotheses of habitat selection during each season (S1 Table).

During winter and spring, females selected habitats that provided increased forage and security, moderated thermal conditions, and lowered energetic costs of movement. During these seasons, the synoptic model that included habitat variables associated with all four ecological hypotheses best fit the data (Table 2; S2 Text). Distance to escape terrain was negatively associated with selected habitat inferring that females were choosing habitat near escape terrain (Table 3, S3–S5 Tables). This habitat variable had the highest influence on selection in comparison to all other habitat and climate variables during winter and spring but was significantly selected only during spring. During winter and spring, percent cover of shrub/scrub, slope, and solar radiation were statistically significant with sheep selecting areas with lower percent cover of shrub/scrub, greater slope, and higher solar radiation. For spring, some climate variables (i.e., snow depth and snow extent) were included in >1 ecological hypothesis; snow was hypothesized to influence both nutrition by limiting access to forage and movement by affecting energetic costs of locomotion. Therefore, although the global model, including both the nutrition and movement hypotheses, was best supported by the data, we were not able to clearly distinguish between support for the ecological mechanisms by which snow might influence habitat selections during spring.

**Table 2:** Habitat and climate variables in the best supported ecological hypotheses to explain habitat selection during 3 seasons by female Dall’s sheep (*Ovis dalli dalli*) in Lake Clark National Park and Preserve, Alaska, USA. Variable group, habitat and climate variables, positive or negative effects on selection, season (winter, spring, or summer), ecological hypotheses (Security + Thermal + Movement + Nutrition or Security + Nutrition), and number of parameters (K) are shown. North, South, or both refers to the distinct regions where radio-collared females occurred (see Fig 2). See S1 Table for a description of each habitat and climate variable and S2 Text for variable reduction and model selection steps.

Group	Habitat or climate variable	Winter	Spring	Summer
		Security + Thermal + Movement + Nutrition	Security + Thermal + Movement + Nutrition	Security + Nutrition
		K = 12	K = 12	K = 7
Vegetative	alpine dwarf scrub^a^	- (North), + (South, both)	+	+ (North),—(South, both)
	NDVI^b^	+	+	+
	shrub/scrub^a^	-	-	-
Topographic	distance to escape terrain	-	-	-
	elevation	+	+	+
	mean slope x ruggedness^c^	+	N/A	+
	ruggedness^d^	N/A	- (North), + (South, both)	N/A
	sine of aspect^e^	- (North, both), + (South)	N/A	N/A
	slope	+	+	- (North, both), + (South)
Climate	air temperature	+	+	N/A
	snow depth	- (North, both), + (South)	-	N/A
	snow extent^a^	N/A	- (North, both), + (South)	N/A
	solar radiation index	+	+	N/A
	wind speed	+	+	N/A

^a^percent area evaluated within 270-m radius circular buffer

^b^normalized difference vegetation index

^c^evaluated across 3x3 pixels at 30-m resolution, this is an interaction variable

^d^evaluated across 7x7 pixels at 90-m resolution based on Sappington et al. [45]

^e^an index of eastness

**Table 3:** Mean parameter estimates and *P*-values from Student’s t-test (significant values of ≤0.05 are shown in bold) for habitat and climate variables used to assess seasonal habitat selection by female Dall’s sheep (*Ovis dalli dalli*) in Lake Clark National Park and Preserve, Alaska, USA. Mean parameter estimates and standard errors are calculated across individuals for the North, South, and both regions (See Fig 2). The parameter estimates are standardized based on the per standard deviation unit change in the original habitat variable and can be compared across habitat variables. The t-test evaluated the significance of each individual parameter estimate as different from zero. See S3–S5 Tables for parameter estimates and standard errors by individual.

Season	Habitat or climate variable	North			South			Both		
		Mean parameter estimate	Mean standard error	*P*-value	Mean parameter estimate	Mean standard error	*P*-value	Mean parameter estimate	Mean standard error	*P*-value
Winter	alpine dwarf scrub^a^	-0.02	0.64	0.97	2.76	0.79	0.13	1.09	0.70	0.17
	NDVI^b^	3.01	1.35	0.06	3.40	2.37	0.27	**3.17**	**1.75**	**0.03**
	shrub/scrub^a^	-3.28	1.34	0.07	**-3.08**	**1.10**	**0.03**	**-3.20**	**1.24**	**0.01**
	snow depth	**-5.31**	**4.06**	**0.05**	0.72	2.03	0.63	-2.90	3.25	0.1
	distance to escape terrain	-9.81	21.42	0.48	-21.83	21.74	0.1	-14.62	21.55	0.12
	elevation	2.57	8.83	0.46	8.11	6.78	0.2	4.79	8.01	0.12
	mean slope x ruggedness^c^	1.55	2.84	0.5	3.57	4.48	0.51	2.36	3.49	0.33
	slope	**1.40**	**1.08**	**0.01**	0.59	1.10	0.52	**1.08**	**1.09**	**0.02**
	sine of aspect^d^	-0.34	0.21	0.06	0.19	0.25	0.32	-0.13	0.23	0.33
	air temperature	0.18	8.64	0.95	11.77	8.69	0.17	4.82	8.66	0.2
	solar radiation index	**0.02**	**0.01**	**0.03**	0.01	0.01	0.15	**0.01**	**0.01**	**0.01**
	wind speed	0.08	0.10	0.56	0.62	0.35	0.24	0.30	0.20	0.17
Spring	alpine dwarf scrub^a^	**1.39**	**0.44**	**<0.01**	**0.68**	**0.60**	**0.04**	**1.07**	**0.51**	**<0.01**
	NDVI^b^	0.48	0.87	0.39	0.29	1.31	0.72	0.39	1.07	0.4
	shrub/scrub	**-2.36**	**1.15**	**0.01**	**-3.01**	**1.15**	**<0.01**	**-2.66**	**1.15**	**<0.01**
	snow extent^a^	-0.48	0.52	0.12	0.12	0.66	0.74	-0.21	0.58	0.37
	distance to escape terrain	**-47.02**	**16.33**	**<0.01**	**-27.10**	**16.93**	**<0.01**	**-37.92**	**16.61**	**<0.01**
	elevation	4.46	9.30	0.32	**11.95**	**9.66**	**0.01**	**7.88**	**9.46**	**0.01**
	ruggedness^e^	-0.46	1.42	0.59	1.48	1.16	0.06	0.43	1.30	0.46
	slope	**1.82**	**1.09**	**<0.01**	**2.49**	**1.19**	**<0.01**	**2.13**	**1.13**	**<0.01**
	snow depth	**-2.96**	**2.29**	**<0.01**	**-2.04**	**1.94**	**0.03**	**-2.54**	**2.13**	**<0.01**
	air temperature	1.75	9.07	0.72	**11.30**	**10.54**	**0.01**	6.12	9.74	0.06
	solar radiation index	**0.95**	**0.42**	**<0.01**	**1.32**	**0.54**	**<0.01**	**1.12**	**0.47**	**<0.01**
	wind speed	0.07	0.12	0.32	0.01	0.15	0.9	0.04	0.13	0.44
Summer	alpine dwarf scrub^a^	**0.74**	**0.37**	**0.01**	**-1.49**	**1.00**	**0.05**	-0.24	0.65	0.53
	NDVI^b^	0.32	0.47	0.28	0.16	0.90	0.78	0.25	0.66	0.41
	shrub/scrub^a^	**-3.27**	**1.32**	**0.01**	**-5.68**	**4.90**	**0**	**-4.33**	**2.90**	**<0.01**
	distance to escape terrain	**-41.88**	**12.99**	**<0.01**	**-14.73**	**11.13**	**0.04**	**-29.90**	**12.17**	**<0.01**
	elevation	**4.29**	**1.43**	**0.01**	**6.62**	**1.50**	**<0.01**	**5.32**	**1.46**	**<0.01**
	mean slope x ruggedness^c^	0.27	1.25	0.76	**4.34**	**2.50**	**0.01**	**2.07**	**1.80**	**0.03**
	slope	-0.39	0.79	0.16	0.30	0.80	0.45	-0.09	0.79	0.71

^a^percent area evaluated within 270-m radius circular buffer

^b^normalized difference vegetation index

^c^evaluated across 3x3 pixels at 30-m resolution, this is an interaction variable

^d^an index of eastness

^e^evaluated across 7x7 pixels at 90-m resolution based on Sappington et al. [45]

During winter, Dall’s sheep selected habitat where forage was likely to be more accessible. Selected areas had steep slopes, high windspeeds, higher NDVI values, and higher solar radiation (Table 3). Because NDVI values were available during winter, we were able to assess that sheep were selecting windswept areas that provided access to green vegetation. We noted significant selection for areas with shallow snow in the North region, but not in the South region, possibly because data were available only for one year (Table 3).

As expected during spring, Dall’s sheep selected habitat characteristics that would provide both security and thermal shelter for lambs. They used areas near escape terrain with steep slopes at high elevations. These areas also had higher windspeeds and shallower snow. Despite the higher winds, sheep also selected sites with warmer temperatures and more solar radiation (Table 3). In addition, females were more likely to select areas with higher NDVI values, likely reflecting green vegetation exposed by wind and solar radiation. Dall’s sheep also avoided shrub/scrub vegetation, suggesting that the green vegetation likely represented low-growing grasses, sedges, and herbs, which are characteristic of alpine dwarf scrub. The direction of these relationships was consistent across regions, but selection for higher elevation habitats with warmer air temperatures was statistically significant only in the South region (Table 3).

During summer as hypothesized, our ecological hypotheses for nutritional needs and security were shown to influence habitat selection (Table 2). Females selected rugged, steep areas higher in elevation close to escape terrain and avoided areas with shrub/scrub vegetation (Table 3). The interaction between mean slope and ruggedness had a significant and positive relationship, indicating that females selected areas that were both rugged and steep (Table 3). Similar to winter and spring, the distance to escape terrain variable had the greatest influence on habitat section compared to the other habitat and climate variables.

## Discussion

We tested ecological hypotheses of nutritional needs, security from predators, energetic costs of movement, and thermal shelter to provide a foundation for understanding and anticipating how environmental change may influence habitat selection by Dall’s sheep under future climates. We defined ecological hypotheses based on resource needs and then competed combinations of habitat and climate variables to parameterize models that best represented each of the ecological requirements in each season. Our quantitative modeling approach facilitated testing broad, *a priori* ecological hypotheses while allowing the data to direct selection of specific variables underlying each ecological hypothesis, and importantly, this approach provided an ecological context for assessing how climate affects habitat selection.

We found that seasonal habitat selection by Dall’s sheep in LCNPP was influenced by multiple ecological requirements simultaneously. Across all seasons, sheep selected steep rugged areas near escape terrain where they could more readily detect, escape, or out-maneuver predators. Additionally, sheep avoided areas with taller shrubs where visibility of the surrounding environment would likely reduce their ability to visually detect predators (Table 3). Forage variables (e.g., alpine dwarf shrub) also were selected across seasons, and during winter sheep used windy, steep slopes (Table 3). In addition, we documented that climate variables influenced habitat selection through both direct and potentially indirect pathways. During spring and winter, sheep selected habitat with warmer air temperatures and lower snow depths, conditions that likely reduce energy expenditure via moderation of the thermal environment and reduction of energetic costs of movement [35, 39, 66]. Indirectly, climate is linked to the expansion of shrubs in alpine regions of southern Alaska [12], and shrub/scrub vegetation was significantly avoided by sheep during all seasons. The high latitude alpine environments, to which Dall’s sheep are well adapted, are currently experiencing rapid climate change, which may be linked to population declines of this iconic species [25, 28, 30, 31]. Our results suggest multiple pathways by which changes in the climate could influence availability and distribution of suitable habitat for Dall’s sheep.

Our findings are consistent with previous qualitative assessments of habitat selection by Dall’s sheep [18, 40], as well as recent quantitative analyses [22, 25, 37, 38, 41], however, we used data from daily movements (i.e., every 5–7 hours) of Dall’s sheep to quantify parameter estimates of seasonal habitat selection. Because our analysis used daily time-steps within seasons, we could incorporate climate variables (e.g., snow, wind, and temperature) that can change over short time intervals. This approach provided a fine temporal resolution often lacking in seasonal studies of resource selection [49, 67, 68]. It also creates a foundation for wildlife managers to understand how changes in weather variables associated with broader patterns of climate change might affect future distribution of suitable habitats available to Dall’s sheep in LCNPP.

### Ecological hypotheses of seasonal habitat selection

We anticipated that energy conservation would be a strong ecological requirement of habitat selection by Dall’s sheep during winter when harsh weather conditions and limited forage availability create energetic challenges. This combination of stressors can contribute to lower survival and affect subsequent reproduction [17, 33, 69]. Supporting this expectation, we documented that sheep selected areas with lower snow depth, higher solar radiation, and warmer air temperatures with air temperature having a greater influence on selection than either snow depth or solar radiation (Table 3). Higher air temperatures aid in maintaining internal body temperature, which conserves energy. Snow depth likely directly affects energetics because energy expenditure of locomotion by ungulates increases with snow depth especially when snow depth exceeds chest height [10, 44, 70]. Indeed, Mahoney et al. [35] documented that Dall’s sheep at LCNPP selected areas with snow depths ≤ 25 cm, which is about half of their average chest height, and selection declined sharply at snow depths >25 cm. Sheep also selected areas during winter with higher wind speeds, which typically have less snow that not only reduces movement costs, but also provides access to low-growing forage indicated by positive NDVI values [18, 35, 37, 40].

In contrast to winter, we expected that habitat selection during the spring would be determined by resource needs associated with reproduction, including both security and thermal shelter for lambs. Indeed, sheep not only selected sites with steep and rugged terrain, but they also used habitats that were warmer and received relatively greater amounts of solar radiation. Access to forage, such as grasses, sedges, and herbs, also was important in habitat selected during the spring when energetic demands are elevated due to gestation and lactation (Table 3). A similar strategy of foraging near escape terrain during spring has been documented for female mountain goats (*Oreamnos americanus* [56]), Stone sheep [22], mouflon (*Ovis gmelini* [71]), and bighorn sheep (*Ovis canadensis* [72]). Our findings indicated that Dall’s sheep in both regions of LCNPP appeared to balance security from predators with nutritional needs by selecting habitats that provided both in proximity.

During summer, we expected that forage availability would be an important factor in habitat selection because of elevated energetic requirements due to lactation, lamb growth, and recuperation of maternal body condition. We documented significant selection for alpine areas with alpine dwarf scrub vegetation, which includes high-quality forages, such as grasses, sedges, herbs, and alpine dwarf evergreen shrubs [73–75]. Similar areas also were selected by Dall’s sheep in Wrangell-St. Elias National Park and Preserve and the western Chugach Mountains in southcentral Alaska as well as by Stone sheep in the northern Rocky Mountains of British Columbia [38, 41, 74]. Furthermore, an index of green vegetation (NDVI) was positively, but not significantly selected by sheep during summer in LCNPP (Table 3). Similar patterns were reported by van de Kerk et al. [34] who found that maximum summer NDVI was associated with higher lamb survival and Roffler et al. [38] who noted that Dall’s sheep selected intermediate values of NDVI during summer. Mountain sheep move to forage in areas with newly emerged green vegetation with high nutritional content [18, 74], and the lack of a strong effect of NDVI on habitat selection during summer could be related to the presence of alpine dwarf evergreen scrub year round [73] and/or to tradeoffs associated with other ecological needs including security [19].

Because lambs likely remain vulnerable to predation throughout the summer, we also expected females to select habitats with terrain that provided security from potential predators. As expected, high-elevation areas that were close to escape terrain were selected by females during summer (Table 3). However, they did not consistently select the steepest slopes, which might indicate use of moderately rugged terrain to facilitate mobility for their lambs [38, 41]. Use of areas in close proximity to escape terrain is a common strategy employed by female mountain ungulates with young, including mountain goats, which spent >60% of their time foraging within 80 m of escape terrain [56, 76].

We used the best data available for evaluating habitat selection by Dall’s sheep at LCNPP, but we recognize limitations in our data and analysis. First, Dall’s sheep, like all animals, select habitat across a diversity of spatial and temporal scales [35]. Although we competed parameters that reflected habitat within different-sized buffers, we evaluated habitat selection at a single spatio-temporal scale (i.e., a resolution of 90 m at a daily time step) because of computational constraints. Analyses using the synoptic model at the daily time scale across the large spatial extent of LCNPP were computationally challenging, even with access to the NASA cloud computing resources. However, the availability of GPS telemetry data provided a fine temporal resolution for the location data, which allowed us to evaluate selection of climate variables that varied over short time intervals (e.g., snow depth). Indeed, evaluation of the influence of climate variables on habitat selection requires nearly real-time data for both use and availability of habitat resources. Second, our spatial habitat variables were based on or derived from remotely sensed data, which are representations of reality that contain inherent spatial errors. To minimize the spatial errors, we resampled 30-m data to 90 m and generalized the land cover classes from more specific ones [50]. Third, the SnowModel results can often be improved with model training data, however, weather data (e.g., snow depth and air temperature) at high elevation sites within the North and South regions in LCNPP were limited (S1 Text), which is a common challenge in arctic and subarctic environments [77]. Inclusion of data from additional weather stations would help improve estimates of the climate variables. Nonetheless, our SnowModel simulations were consistent with observed data used for model training and were reliable at fine spatial scales in our study region [35]. Lastly, our models tested for directional changes in selection of habitat and climate variables, in part, because multiple variables and scales considered simultaneously precluded us from increasing the number of variables by adding nonlinear relationships (S1 Table). However, we believe that such relationships are useful to consider because Dall’s sheep have been shown to use mid-elevations and intermediate values of NDVI [38].

### Direct and indirect climate effects

The daily resolution of our telemetry data and the daily estimates of climate variables enabled us to examine how individuals selected habitat in response to climate properties, including snow depth and extent, wind speed, and air temperature. Unlike seasonal models of resource selection, this approach provides a mechanistic foundation for asking how sheep might respond to directional changes in climate over time. As the arctic and subarctic climate warms, changes in precipitation, including snow depth and extent, could have direct effects on Dall’s sheep movements and habitat use [4, 35, 46]. Female sheep in LCNPP selected habitat with less snow cover and shallower snow depths during spring and winter, and warmer winters will reduce both of these factors, allowing more and longer access to forage [4, 8, 33]. However, changes in the composition and frequency of precipitation, such as rain-on-snow events and changes in temperature that lead to a higher frequency of freeze-thaw cycles, will increase the cost of movement by sheep and reduce access to forage [9, 33, 34, 67]. Because of its location near the southern coast of Alaska, LCNPP could be particularly influenced by rain-on-snow events and a higher frequency of freeze-thaw cycles as opposed to regions in the interior of Alaska [9, 34, 78].

In addition, changes in phenology affect reproduction and forage availability during spring when nutritional needs of females are elevated [33, 79]. Reproduction by Dall’s sheep corresponds with spring green up, and changes in phenology affect timing and synchrony of births and subsequent lambing behaviors [49, 80]. Recent analyses indicated that the date of snow disappearance in spring, which is inversely related to spring green up, was the best predictor of recruitment in Dall’s sheep populations across their range [33]. Indeed, a single year with a late spring can impact recruitment leading to population declines with recovery taking several years [32, 33]. In contrast, advanced onset of spring will extend the growing season and prolong access to forage helping Dall’s sheep to meet the nutritional demands of gestation and lactation [17, 33]. However, warmer and longer growing seasons may not benefit sheep as expected because earlier plant senescence (and hence lower forage quality) is linked to warmer temperatures [4].

Potential indirect effects of climate change on Dall’s sheep include expansion of shrub habitats in arctic and subarctic regions, which is linked to a warming climate [12, 13, 61, 81–83]. Shrub/scrub areas, which are dominated by relatively tall shrubs with >20% canopy, were consistently avoided by female Dall’s sheep in LCNPP during all seasons (Table 3). Shrub expansion includes infilling of existing shrub cover, increases in shrub height, and an advancing shrub line, which intrudes into existing vegetation cover [84]. Such changes decrease and fragment preferred forage in alpine areas, thereby constraining habitat used by Dall’s sheep [12]. If sheep avoid shrub/scrub in part because of reduced ability to detect predators, then increased shrub expansion is likely indicative of an increase in predation risk [25, 54]. Furthermore, predation risk may be higher during spring when females and their young are most vulnerable, and indeed, mortality of sheep in LCNPP was highest during spring [85]. Spring is a critical period for sheep survival and recruitment, and shrub expansion influences both demographic processes.

## Conclusions

High-latitude alpine regions are changing in ways that are and will continue to affect distribution and quality of habitat for Dall’s sheep. Widespread population declines of Dall’s sheep have raised concerns about their ability to adapt to current and future changes in climate and distribution of resources. Testing multiple ecological hypotheses related to habitat requirements (i.e., nutrition, security, thermal shelter, energy conservation) for Dall’s sheep indicated that they balance resource selection to meet multiple needs across seasons and such behaviors are likely fine-tuned to long-term patterns of phenology and climate. Constraints imposed by direct and indirect effects of a changing climate could limit their ability to continue to balance resource needs and lead to continued population declines. However, several management approaches could help to mitigate these constraints and promote resiliency of alpine habitats. Maintaining connectivity across multiple habitat types would allow movement among patches of suitable habitat, managing landscapes undergoing change to conserve ecosystem structure and function into the future, and expanding stakeholder involvement to co-manage these natural resources for the future are options for promoting resiliency of the alpine habitats that support Dall’s sheep populations [86, 87].

## Supporting information

S1 Fig Example plots of GPS location data for distances from capture release sites for individual female Dall’s sheep (*Ovis dalli dalli*) in Lake Clark National Park and Preserve, Alaska, USA, during a) 2007, b) 2008, and c) across both years. (PDF)

S1 Table Climate, topographic, and vegetative variables as well as interaction terms used for development of models of habitat selection analysis for female Dall’s sheep (*Ovis dalli dalli*) in Lake Clark National Park and Preserve, Alaska, USA during 2006 and 2007. (PDF)

S2 Table Ecological hypotheses and combinations of ecological hypotheses for each season that were evaluated using a synoptic model to assess habitat selection of female Dall’s sheep in Lake Clark National Park and Preserve, Alaska, USA during 2006–2007. (PDF)

S3 Table Parameter estimates and standard errors (SE) for each habitat and climate variable in the best supported model for winter by individual female Dall’s sheep. (PDF)

S4 Table Parameter estimates and standard errors (SE) for each habitat and climate variable in the best supported model for spring by individual female Dall’s sheep. (PDF)

S5 Table Parameter estimates and standard errors (SE) for each habitat and climate variable in the best supported model for summer by individual female Dall’s sheep. (PDF)

S1 Text Summary of SnowModel simulation. (PDF)

S2 Text Methods for variable reduction and selection of seasonal models. (PDF)
