# Supplementary material for: Habitat selection by Dall’s sheep is influenced by multiple factors including direct and indirect climate effects
Source: PLoS One. 2021 Mar 18;16(3):e0248763. doi: 10.1371/journal.pone.0248763 (PMC7971871; doi:10.1371/journal.pone.0248763)

S1 Figure. Example plots of GPS location data for distances from capture release sites for individual female Dall's sheep (*Ovis dalli dalli*) in Lake Clark National Park and Preserve, Alaska, USA, during a) 2007, b) 2008, and c) across both years. Seasonal movements between winter and summer periods were evident and consistent across years reflecting distinct seasonal shifts in space use that guided decisions about partitioning the annual period into seasons for habitat analyses based on movement.

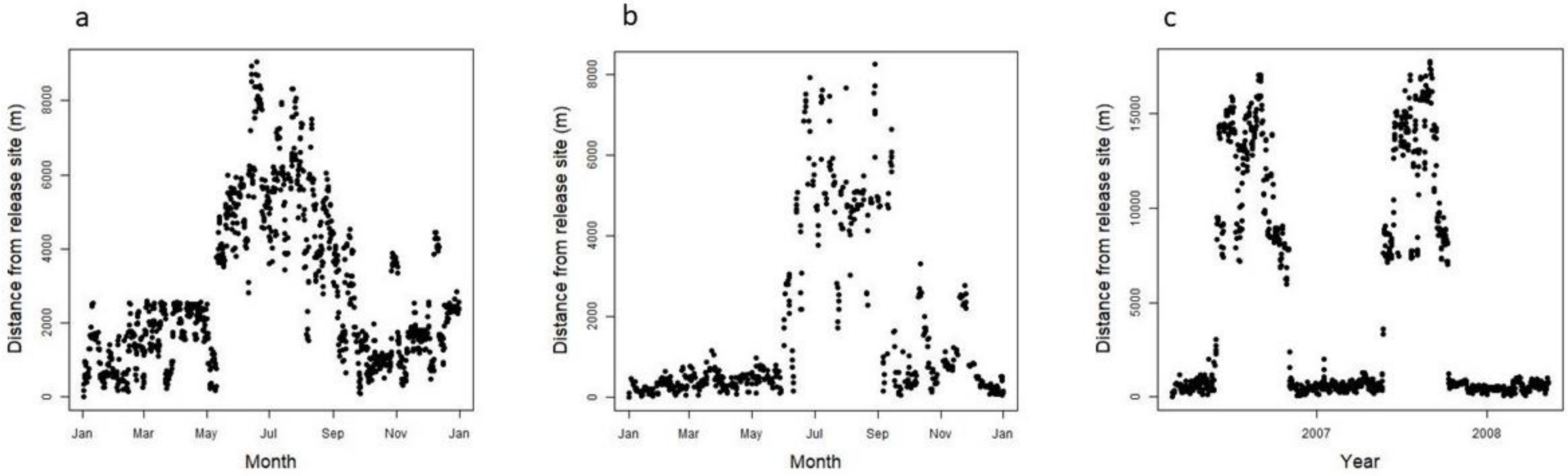

Supplement: S1 Fig — (PDF) [file pone.0248763.s001.pdf]
