# Supplementary material for: Habitat selection by Dall’s sheep is influenced by multiple factors including direct and indirect climate effects
Source: PLoS One. 2021 Mar 18;16(3):e0248763. doi: 10.1371/journal.pone.0248763 (PMC7971871; doi:10.1371/journal.pone.0248763)
Supplement: S1 Table — (PDF) [file pone.0248763.s002.pdf]

S1 Table. Climate, topographic, and vegetative variables as well as interaction terms used for development of models of habitat selection analysis for Dall's sheep females (*Ovis dalli dalli*) in Lake Clark National Park and Preserve, Alaska, USA during 2006 and 2007. Variables in bold text were retained in our habitat selection analysis after omitting model variables that had an r value ≥ 0.7. These variables were used for the North and South regions.

Group	Variable name	Description	Unit of measure	Data type	Data source
**Climate**	**Air temperature**	**Ambient temperature of air based on SnowModel**	**degees Celsius**	**Continuous**	**Liston and Elder (2006)**
Climate	Percent of snow extent within 135-meters	Percent of snow cover in circular plot with 135-meter radius	percent	Continuous	Liston and Elder (2006)
**Climate**	**Percent of snow extent within 270-meters**	**Percent of snow cover in circular plot with 270-meter radius**	**percent**	**Continuous**	**Liston and Elder (2006)**
**Climate**	**Snow depth**	**Depth of snow based on SnowModel**	**centimeters**	**Continuous**	**Liston and Elder (2006)**
Climate	Snow extent	Extent of snow coverage based on SnowModel	90-meter pixel	Categorical	Liston and Elder (2006)
**Climate**	**Solar radiation index**	**Daily values for solar radiation during 2006-2008**	**N/A**	**Continuous**	**Rich et al. (1994), Fu and Rich (2002), Keating et al. (2007)**
**Climate**	**Wind speed**	**Speed of wind based on SnowModel**	**meters per second**	**Continuous**	**Liston and Elder (2006)**
Interaction term	Distance to escape terrain and barren areas	Interaction between distance to escape terrain, which was defined as areas with slopes >30 degrees and pixels classified as barren areas in National Land Cover Dataset	N/A	Continuous	USGS National Elevation Dataset (NED) https://lta.cr.usgs.gov/NED and National Land Cover Dataset (http://www.mrlc.gov/finddata.php)
Interaction term	Distance to escape terrain and percent barren areas within 135 meters	Interaction between distance to escape terrain, which was defined as areas with slopes >30 degrees and percent barren cover in circular plot with 135-meter radius	N/A	Continuous	USGS National Elevation Dataset (NED) https://lta.cr.usgs.gov/NED and National Land Cover Dataset (http://www.mrlc.gov/finddata.php)
Interaction term	Distance to escape terrain and percent barren areas within 270 meters	Interaction between distance to escape terrain, which was defined as areas with slopes >30 degrees and percent barren cover in circular plot with 270-meter radius	N/A	Continuous	USGS National Elevation Dataset (NED) https://lta.cr.usgs.gov/NED and National Land Cover Dataset (http://www.mrlc.gov/finddata.php)
**Interaction term**	**Mean Slope and ruggedness interaction variable**	**Mean slope X ruggedness at 3X3 pixel window**	**N/A**	**Continuous**	**Sappington et al. (2007) and USGS National Elevation Dataset (NED) https://lta.cr.usgs.gov/NED**
Interaction term	Mean Slope and ruggedness interaction variable	Mean slope X ruggedness at 5X5 pixel window	N/A	Continuous	Sappington et al. (2007) and USGS National Elevation Dataset (NED) https://lta.cr.usgs.gov/NED
Interaction term	Mean Slope and ruggedness interaction variable	Mean slope X ruggedness at 7X7 pixel window	N/A	Continuous	Sappington et al. (2007) and USGS National Elevation Dataset (NED) https://lta.cr.usgs.gov/NED
Interaction term	Standard deviation of slope and ruggedness interaction variables	Standard deviation of slope X ruggedness at 3X3 pixel window	N/A	Continuous	Sappington et al. (2007) and USGS National Elevation Dataset (NED) https://lta.cr.usgs.gov/NED
Interaction term	Standard deviation of slope and ruggedness interaction variables	Standard deviation of slope X ruggedness at 5X5 pixel window	N/A	Continuous	Sappington et al. (2007) and USGS National Elevation Dataset (NED) https://lta.cr.usgs.gov/NED
Interaction term	Standard deviation of slope and ruggedness interaction variables	Standard deviation of slope X ruggedness at 7X7 pixel window	N/A	Continuous	Sappington et al. (2007) and USGS National Elevation Dataset (NED) https://lta.cr.usgs.gov/NED
Topographic	Cosine of aspect	Converts values of aspect into an index of north-south values (i.e., northness)	N/A	Continuous	USGS National Elevation Dataset (NED) https://lta.cr.usgs.gov/NED
**Topographic**	**Distance to escape terrain**	**Distance to escape terrain, which was defined as areas with slopes >30 degrees**	**meter**	**Continuous**	**USGS National Elevation Dataset (NED) https://lta.cr.usgs.gov/NED**
**Topographic**	**Elevation**	**Elevation above sealevel, 10 m intervals**	**meter**	**Continuous**	**USGS National Elevation Dataset (NED) https://lta.cr.usgs.gov/NED**
Topographic	Ruggedness	Index of ruggedness at 3X3 pixel window	N/A	Continuous	Sappington et al. (2007)
Topographic	Ruggedness	Index of ruggedness at 5X5 pixel window	N/A	Continuous	Sappington et al. (2007)
**Topographic**	**Ruggedness**	**Index of ruggedness at 7X7 pixel window**	**N/A**	**Continuous**	**Sappington et al. (2007)**
**Topographic**	**Sine of aspect**	**Converts values of aspect into an index of east-west values (i.e., eastness)**	**N/A**	**Continuous**	**USGS National Elevation Dataset (NED) https://lta.cr.usgs.gov/NED**
**Topographic**	**Slope**	**Ratio of vertical distance to horizontal distance across a landscape**	**degrees**	**Continuous**	**USGS National Elevation Dataset (NED) https://lta.cr.usgs.gov/NED**
Vegetation	Barren area	Pixels classified as barren areas in National Land Cover Dataset	30-meter pixel	Categorical	National Land Cover Dataset (http://www.mrlc.gov/finddata.php)
Vegetation	Deciduous forest	Pixels classified as deciduous forest in National Land Cover Dataset	30-meter pixel	Categorical	National Land Cover Dataset (http://www.mrlc.gov/finddata.php)
Vegetation	Alpine dwarf scrub	Pixels classified as alpine dwarf scrub in National Land Cover Dataset	30-meter pixel	Categorical	National Land Cover Dataset (http://www.mrlc.gov/finddata.php)
Vegetation	Evergreen forest	Pixels classified as evergreen forest in National Land Cover Dataset	30-meter pixel	Categorical	National Land Cover Dataset (http://www.mrlc.gov/finddata.php)
Vegetation	Ice	Pixels classified as ice in National Land Cover Dataset	30-meter pixel	Categorical	National Land Cover Dataset (http://www.mrlc.gov/finddata.php)
Vegetation	Mixed forest	Pixels classified as mixed forest in National Land Cover Dataset	30-meter pixel	Categorical	National Land Cover Dataset (http://www.mrlc.gov/finddata.php)
**Vegetation**	**Normalized difference vegetation index**	**Index of maximum normalized difference vegetation index capture weekly, a measure of vegetation green-up**	**30-meter pixel**	**Continuous**	**MODIS, www.gina.alaska.edu**
Vegetation	Percent barren cover within 135 meters	Percent barren cover in circular plot with 135-meter radius	percent	Continuous	National Land Cover Dataset (http://www.mrlc.gov/finddata.php)
Vegetation	Percent barren cover within 270 meters	Percent barren cover in circular plot with 270-meter radius	percent	Continuous	National Land Cover Dataset (http://www.mrlc.gov/finddata.php)
Vegetation	Percent deciduous forest cover wihtin 135 meters	Percent deciduous forest cover in circular plot with 135-meter radius	percent	Continuous	National Land Cover Dataset (http://www.mrlc.gov/finddata.php)
Vegetation	Percent deciduous forest cover within 270 meters	Percent deciduous forest cover in circular plot with 270-meter radius	percent	Continuous	National Land Cover Dataset (http://www.mrlc.gov/finddata.php)
Vegetation	Percent alpine dwarf scrub cover within 135 meters	Percent alpine dwarf scrub cover in circular plot with 135-meter radius	percent	Continuous	National Land Cover Dataset (http://www.mrlc.gov/finddata.php)

**Vegetation**	**Percent alpine dwarf scrub cover wtihin 270 meters**	**Percent alpine dwarf scrub cover in circular plot with 270-meter radius**	**percent**	**Continuous**	**National Land Cover Dataset (http://www.mrlc.gov/finddata.php)**
Vegetation	Percent evergreen forest cover within 135 meters	Percent evergreen forest cover in circular plot with 135-meter radius	percent	Continuous	National Land Cover Dataset (http://www.mrlc.gov/finddata.php)
Vegetation	Percent evergreen forest cover within 270 meters	Percent evergreen forest cover in circular plot with 270-meter radius	percent	Continuous	National Land Cover Dataset (http://www.mrlc.gov/finddata.php)
Vegetation	Percent ice cover within 135 meters	Percent ice cover in circular plot with 135-meter radius	percent	Continuous	National Land Cover Dataset (http://www.mrlc.gov/finddata.php)
Vegetation	Percent ice cover within 270 meters	Percent ice cover in circular plot with 270-meter radius	percent	Continuous	National Land Cover Dataset (http://www.mrlc.gov/finddata.php)
Vegetation	Percent mixed forest cover within 135 meters	Percent mixed forest cover in circular plot with 135-meter radius	percent	Continuous	National Land Cover Dataset (http://www.mrlc.gov/finddata.php)
Vegetation	Percent mixed forest cover within 270 meters	Percent mixed forest cover in circular plot with 270-meter radius	percent	Continuous	National Land Cover Dataset (http://www.mrlc.gov/finddata.php)
Vegetation	Percent shrub/scrub cover within 135 meters	Percent shrub/scrub cover in circular plot with 135-meter radius	percent	Continuous	National Land Cover Dataset (http://www.mrlc.gov/finddata.php)
**Vegetation**	**Percent shrub/scrub cover within 270 meters**	**Percent shrub/scrub cover in circular plot with 270-meter radius**	**percent**	**Continuous**	**National Land Cover Dataset (http://www.mrlc.gov/finddata.php)**
Vegetation	Shrub/scrub	Pixels classified as shrub/scrub areas in National Land Cover Dataset	30-meter pixel	Categorical	National Land Cover Dataset (http://www.mrlc.gov/finddata.php)
