# Supplementary material for: Habitat selection by Dall’s sheep is influenced by multiple factors including direct and indirect climate effects
Source: PLoS One. 2021 Mar 18;16(3):e0248763. doi: 10.1371/journal.pone.0248763 (PMC7971871; doi:10.1371/journal.pone.0248763)
Supplement: S2 Table — (PDF) [file pone.0248763.s003.pdf]

S2 Table. Ecological hypotheses and combinations of ecological hypotheses for each season that were evaluated using a synoiptic model to assess habitat selection of Dall's sheep females (*Ovis dalli dalli*) in Lake Clark National Park and Preserve, Alaska, USA during 2006-2007. Season, ecological hypotheses, habitat variables, number of parameters (i.e., K), AIC values, and delta AIC values are shown. Values for each year, each region, both years, both regions, and all regions and years are shown. A subset of these data are shown in Table 2 of the main text. See S1 Table for more details on each habitat variable. The model that best fit the data for each season is shown in bold. See Fig. 1 and Table 1 in main text for description of ecological hypotheses.

				North 2006		North 2007		South 2006		South 2007		North		South		2006		2007		All regions and years	
Season	Ecological hypotheses and combinations of ecological hypotheses	Habitat variables	K	AIC_c	ΔAIC_c	AIC_c	ΔAIC_c	AIC_c	ΔAIC_c	AIC_c	ΔAIC_c	AIC_c	ΔAIC_c	AIC_c	ΔAIC_c	AIC_c	ΔAIC_c	AIC_c	ΔAIC_c	AIC_c	ΔAIC_c
Spring																					
	Null		0	50066.12	1690.25	46346.11	1917.75	28304.69	1313.47	35615.39	1520.84	96412.23	3608.00	63920.08	2834.31	78370.81	3003.72	81961.50	3438.59	160332.31	6442.31
	Security	elevation + distance to escape terrain + ruggedness (7 x 7 pixels) + slope	4	49023.43	647.55	45331.12	1016.22	27360.10	735.03	34674.99	1118.24	94354.54	1550.32	62035.09	949.31	76383.52	1016.43	80006.10	1483.20	156389.63	2499.63
	Thermal	solar radiation + wind speed + snow depth + air temperature	4	49480.14	1104.27	45849.10	902.76	27657.12	368.88	34948.80	580.43	95329.24	2525.01	62605.92	1520.15	77137.26	1770.17	80797.90	2274.99	157935.16	4045.16
	Movement	snow depth + snow extent (270 m)	2	49908.21	1532.34	46098.56	1670.20	27953.88	962.67	35534.58	1440.03	96006.77	3202.54	63488.46	2402.69	77862.09	2495.00	81633.14	3110.23	159495.23	5605.23
	Nutrition	alpine dwarf scrub (270 m) + shrub/scrub (270 m) + NDVI + snow extent (270 m)	4	49231.25	855.38	45444.57	1420.75	27726.25	665.90	35212.80	854.24	94675.82	1871.59	62939.05	1853.27	76957.50	1590.41	80657.37	2134.46	157614.87	3724.87
	Security + Thermal	elevation + distance to escape terrain + ruggedness (7 x7 pixels)+ slope + solar radiation + wind speed + snow depth + air temperature	8	48709.49	333.62	44895.10	209.26	27216.05	137.77	34244.27	339.40	93604.59	800.37	61460.32	374.55	75925.54	558.45	79139.37	616.46	155064.91	1174.91
	Security + Movement	elevation + distance to escape terrain + ruggedness (7 x 7 pixels) + slope + snow depth + snow extent (270 m)	6	48939.01	563.14	45018.77	965.95	27227.30	639.93	34581.64	1132.67	93957.78	1153.55	61808.93	723.16	76166.31	799.22	79600.40	1077.49	155766.71	1876.71
	Security + Nutrition	elevation + distance to escape terrain + ruggedness (7 x 7 pixels) + slope + alpine dwarf scrub (270 m) + shrub/scrub (270 m) + NDVI + snow extent (270m)	8	48594.78	218.91	44637.62	711.52	27128.99	398.92	34433.95	733.89	93232.40	428.17	61562.94	477.17	75723.77	356.68	79071.57	548.66	154795.34	905.34
	Thermal + Movement	snow depth + snow extent (270 m) + solar radiation + wind speed + air temperature	5	49448.66	1072.78	45717.32	590.41	27532.99	236.08	34938.39	487.08	95165.97	2361.74	62471.38	1385.61	76981.65	1614.56	80655.70	2132.79	157637.35	3747.35
	Thermal + Nutrition	alpine dwarf scrub (270 m) + shrub/scrub (270 m) + NDVI + snow extent (270 m) + solar radiation + wind speed + snow depth + air temperature	8	48968.29	592.42	45139.88	466.74	27390.14	224.83	34828.45	149.72	94108.17	1303.94	62218.59	1132.81	76358.43	991.34	79968.32	1445.42	156326.75	2436.75
	Movement + Nutrition	alpine dwarf scrub (270 m) + shrub/scrub (270 m) + NDVI + snow extent (270 m) + snow depth	5	49206.55	830.68	45394.30	1288.96	27631.15	541.78	35227.23	843.83	94600.86	1796.63	62858.38	1772.61	76837.70	1470.61	80621.53	2098.62	157459.24	3569.24
	Security + Thermal + Movement	elevation + distance to escape terrain + ruggedness (7 x 7 pixels) + slope + snow depth + snow extent (270 m) + solar radiation + wind speed + air temperature	9	48698.85	322.98	44796.41	244.89	27122.20	65.13	34816.29	346.17	93495.26	691.03	61938.49	852.72	75821.06	453.97	79612.69	1089.78	155433.75	1543.75
	Security + Thermal + Nutrition	alpine dwarf scrub (270 m) + shrub/scrub (270 m) + NDVI + snow extent (270 m) + elevation + distance to escape terrain + ruggedness (7 x 7 pixels) + slope + solar radiation + wind speed + air temperature + snow depth	12	48385.98	10.11	44447.34	18.99	27006.10	14.88	34098.57	4.02	92833.33	29.10	61104.67	18.90	75392.08	24.99	78545.91	23.00	153937.99	48.00
	Security + Movement + Nutrition	alpine dwarf scrub (270 m) + shrub/scrub (270 m) + NDVI + snow extent (270 m) + elevation + distance to escape terrain + ruggedness (7 x 7 pixels) + slope + snow depth	9	48550.21	174.34	44673.24	705.81	27056.34	361.79	34440.72	729.52	93223.46	419.23	61497.06	411.29	75606.56	239.47	79113.96	591.05	154720.52	830.52
	Thermal + Movement + Nutrition	alpine dwarf scrub (270 m) + shrub/scrub (270 m) + NDVI + snow extent (270 m) + snow depth + solar radiation + wind speed + air temperature	8	48946.06	570.19	45134.16	368.05	27353.00	130.98	34824.07	721.73	94080.22	1276.00	62177.07	1091.30	76299.07	931.98	79958.23	1435.32	156257.30	2367.30
	**Security + Thermal + Movement + Nutrition**	**alpine dwarf scrub (270 m) + shrub/scrub (270 m) + NDVI + snow extent (270 m) + elevation + distance to escape terrain + ruggedness (7 x 7 pixels) + slope + snow depth + solar radiation + wind speed + air temperature**	**12**	**48375.87**	**0**	**44428.36**	**0**	**26991.22**	**0**	**34094.55**	**0**	**92804.23**	**0**	**61085.77**	**0**	**75367.09**	**0**	**78522.91**	**0**	**153890.00**	**0.00**
Summer																					
	Null		0	75755.57	7845.35	69231.60	1331.63	41040.88	1207.00	52396.06	1789.28	144987.17	9176.98	93436.94	2996.29	116796.45	9052.35	121627.65	3120.92	238424.11	12173.27
	Security	elevation + slope + (mean slope x ruggedness (3 x 3 pixels)) + distance to escape terrian	4	74815.80	6905.57	68339.20	439.23	39930.34	96.46	50764.85	158.08	143154.99	7344.80	90695.19	254.54	114746.14	7002.03	119104.05	597.31	233850.19	7599.34
	Nutrition	alpine dwarf scrub (270 m) + shrub/scrub (270 m) + NDVI	3	75016.63	7106.40	68555.31	655.34	40328.09	494.21	51523.40	916.63	143571.93	7761.75	91851.49	1410.84	115344.72	7600.61	120078.71	1571.97	235423.43	9172.59
	**Security and Nutrition**	**elevation + slope + (mean slope x ruggedness (3 x 3 pixels)) + distance to escape terrian + alpine dwarf scrub (270 m) + shrub/scrub (270 m) + NDVI**	**7**	**67910.22**	**0**	**67899.96**	**0**	**39833.88**	**0**	**50606.77**	**0**	**135810.19**	**0**	**90440.65**	**0**	**107744.10**	**0**	**118506.74**	**0**	**226250.84**	**0.00**
Winter																					
	Null			n/a	n/a	55312.85	1392.35	n/a	n/a	28632.54	840.79	55312.85	1392.35	28632.54	840.79	n/a	n/a	83945.39	2233.14	83945.39	2233.14
	Security	elevation + slope + (mean slope x ruggedness (3 x 3 pixels)) + distance to escape terrain	4	n/a	n/a	54371.99	451.49	n/a	n/a	28170.15	378.399	54371.99	451.49	28170.15	378.40	n/a	n/a	82542.14	829.89	82542.14	829.89
	Thermal	solar radiation + wind speed + snow depth + air temperature	4	n/a	n/a	54664.31	743.812	n/a	n/a	28262.19	470.439	54664.31	743.81	28262.19	470.44	n/a	n/a	82926.50	1214.25	82926.50	1214.25
	Movement	snow depth + sine aspect	2	n/a	n/a	55259.35	1338.85	n/a	n/a	28572.89	781.145	55259.35	1338.85	28572.89	781.15	n/a	n/a	83832.25	2120.00	83832.25	2120.00
	Nutrition	alpine dwarf scrub (270 m) + shrub/scrub (270 m) + NDVI + snow depth	4	n/a	n/a	54665.98	745.475	n/a	n/a	28039.94	248.196	54665.98	745.47	28039.94	248.20	n/a	n/a	82705.92	993.67	82705.92	993.67
	Security + Thermal	elevation + slope + (mean slope x ruggedness (3 x 3 pixels)) + distance to escape terrain + solar radiation + wind speed + snow depth + air temperature	8	n/a	n/a	54349.89	429.393	n/a	n/a	28143.54	351.793	54349.89	429.39	28143.54	351.79	n/a	n/a	82493.43	781.19	82493.43	781.19
	Security + Movement	elevation + slope + (mean slope x ruggedness (3 x 3 pixels)) + distance to escape terrain + snow depth + sine aspect	6	n/a	n/a	54414.10	493.603	n/a	n/a	28200.56	408.806	54414.10	493.60	28200.56	408.81	n/a	n/a	82614.66	902.41	82614.66	902.41
	Security + Nutrition	alpine dwarf scrub (270 m) + shrub/scrub (270 m) + NDVI + snow depth + elevation + slope + (mean slope x ruggedness (3 x 3 pixels)) + distance to escape terrain	8	n/a	n/a	54140.70	220.196	n/a	n/a	27875.89	84.1439	54140.70	220.20	27875.89	84.14	n/a	n/a	82016.59	304.34	82016.59	304.34
	Thermal + Movement	snow depth + sine aspect + solar radiation + wind speed + air temperature	5	n/a	n/a	54732.71	812.207	n/a	n/a	28379.91	588.163	54732.71	812.21	28379.91	588.16	n/a	n/a	83112.62	1400.37	83112.62	1400.37
	Thermal + Nutrition	alpine dwarf scrub (270 m) + shrub/scrub (270 m) + NDVI + snow depth + solar radiation + wind speed + air temperature	7	n/a	n/a	54363.44	442.939	n/a	n/a	27984.04	192.293	54363.44	442.94	27984.04	192.29	n/a	n/a	82347.48	635.23	82347.48	635.23
	Movement + Nutrition	alpine dwarf scrub (270 m) + shrub/scrub (270 m) + NDVI + snow depth + sine aspect	5	n/a	n/a	54769.32	848.824	n/a	n/a	28045.74	253.996	54769.32	848.82	28045.74	254.00	n/a	n/a	82815.07	1102.82	82815.07	1102.82
	Security + Thermal + Movement	elevation + slope + (mean slope x ruggedness (3 x 3 pixels)) + distance to escape terrain + snow depth + sine aspect + solar radiation + wind speed + air temperature	9	n/a	n/a	54310.71	390.214	n/a	n/a	28130.04	338.287	54310.71	390.21	28130.04	338.29	n/a	n/a	82440.75	728.50	82440.75	728.50
	Security + Thermal + Nutrition	alpine dwarf scrub (270 m) + shrub/scrub (270 m) + NDVI + snow depth + elevation + slope + (mean slope x ruggedness (3 x 3 pixels)) + distance to escape terrain + solar radiation + wind speed + air temperature	11	n/a	n/a	53992.65	72.1497	n/a	n/a	27839.57	47.8219	53992.65	72.15	27839.57	47.82	n/a	n/a	81832.22	119.97	81832.22	119.97
	Security + Movement + Nutrition	alpine dwarf scrub (270 m) + shrub/scrub (270 m) + NDVI + snow depth + elevation + slope + (mean slope x ruggedness (3 x 3 pixels)) + distance to escape terrain + sine aspect	9	n/a	n/a	54031.09	110.59	n/a	n/a	27866.33	74.5792	54031.09	110.59	27866.33	74.58	n/a	n/a	81897.42	185.17	81897.42	185.17
	Thermal + Movement + Nutrition	alpine dwarf scrub (270 m) + shrub/scrub (270 m) + NDVI + snow depth + sine aspect + solar radiation + wind speed + air temperature	8	n/a	n/a	54276.80	356.304	n/a	n/a	27979.94	188.193	54276.80	356.30	27979.94	188.19	n/a	n/a	82256.75	544.50	82256.75	544.50
	**Security + Thermal + Movement + Nutrition**	**alpine dwarf scrub (270 m) + shrub/scrub (270 m) + NDVI + snow depth + elevation + slope + (mean slope x ruggedness (3 x 3 pixels)) + distance to escape terrain + sine aspect + solar radiation + wind speed + air temperature**	**12**	**n/a**	**n/a**	**53920.50**	**0**	**n/a**	**n/a**	**27791.75**	**0**	**53920.50**	**0.00**	**27791.75**	**0.00**	**n/a**	**n/a**	**81712.25**	**0**	**81712.25**	**0.00**
