# Supplementary material for: Habitat selection by Dall’s sheep is influenced by multiple factors including direct and indirect climate effects
Source: PLoS One. 2021 Mar 18;16(3):e0248763. doi: 10.1371/journal.pone.0248763 (PMC7971871; doi:10.1371/journal.pone.0248763)
Supplement: S3 Table — (PDF) [file pone.0248763.s004.pdf]

S3 Table. Parameter estimates and standard errors (SE) for each habitat and climate variable in the best supported model for winter by individual Dall's sheep females (*Ovis dalli dalli*). Values are shown for individuals in the North and South regions within Lake Clark National Park and Preserve, Alaska during 2007. See Table 3 in the main text for mean values of parameter estimates and standard errors for each habitat variable. See S1 Table for description of habitat variables.

Individual	elevation ± SE	distance to escape terrain ± SE	slope ± SE	mean slope x ruggedness[a] ± SE	alpine dwarf shrub[b] ± SE	shrub/scrub[b] ± SE	snow depth ± SE	solar radiation index ± SE	air temperature ± SE	wind speed ± SE	NDVI[c] ± SE	sine of aspect[d] ± SE
North region												
613	-9.39 ± 10.56	1.33 ± 43.33	3 ± 1.16	3.49 ± 3.02	-1.22 ± 1.33	-8.59 ± 1.65	-7.79 ± 11.7	0.03 ± 0.01	17.83 ± 14.61	1.02 ± 0.25	4.82 ± 2.21	-0.42 ± 0.23
615	11.92 ± 9.46	36.01 ± 21.46	-0.2 ± 1.05	10.91 ± 3.07	-2.03 ± 0.9	1.82 ± 0.73	-17.97 ± 6.53	0.04 ± 0.01	0.82 ± 8.82	0.2 ± 0.12	2.5 ± 1.51	-1.19 ± 0.2
616	3.32 ± 9.72	36.86 ± 21.23	1.65 ± 1.15	-2.84 ± 2.21	0.6 ± 0.7	2 ± 0.76	-8.3 ± 6.48	0 ± 0.01	-3.19 ± 8.94	0.33 ± 0.11	3.01 ± 1.54	-0.56 ± 0.22
620	3.32 ± 9.05	26.54 ± 23.81	0.74 ± 1.13	4.65 ± 5.18	2.42 ± 0.64	-12.04 ± 3.51	5.85 ± 1.97	0.02 ± 0.02	-5.91 ± 8.97	-0.27 ± 0.14	10.52 ± 1.73	-0.36 ± 0.21
622	18.56 ± 9.93	-17.07 ± 25.27	1.82 ± 1.26	-11.57 ± 4.28	-2.64 ± 0.61	-4.27 ± 0.88	-10.47 ± 3.1	-0.02 ± 0.01	5.14 ± 9.38	0.1 ± 0.08	6.35 ± 1.75	-0.18 ± 0.22
623	-4.64 ± 8.41	-43.02 ± 12.59	0.94 ± 0.95	0.03 ± 2.5	0.98 ± 0.38	-2.84 ± 1	2.09 ± 1.36	0.02 ± 0.01	-4.36 ± 7.35	-0.17 ± 0.06	1.06 ± 0.93	0.45 ± 0.21
624	12.13 ± 4	-78.3 ± 15.59	-0.42 ± 0.86	2.43 ± 1.07	-0.23 ± 0.46	-4.16 ± 1.09	-1.88 ± 1.67	0.02 ± 0.01	7.12 ± 3.21	-0.02 ± 0.05	4.24 ± 0.49	-0.27 ± 0.21
626	-3.71 ± 7.38	-7.78 ± 15.02	2.21 ± 0.92	-0.92 ± 1.76	0.81 ± 0.32	-2.88 ± 1.88	-6.04 ± 1.63	0 ± 0.01	-4.45 ± 6.5	-0.23 ± 0.05	-2.77 ± 0.95	0.09 ± 0.18
627	-8.36 ± 11	-42.85 ± 14.44	2.91 ± 1.28	7.81 ± 2.47	1.12 ± 0.47	1.42 ± 0.54	-3.26 ± 2.07	0.03 ± 0.01	-11.36 ± 9.96	-0.2 ± 0.05	-2.62 ± 1.01	-0.67 ± 0.22
South region												
501	28.36 ± 5.8	-30.3 ± 9.4	-0.37 ± 0.85	18.46 ± 4.22	1.8 ± 0.37	1.4 ± 0.48	6.05 ± 1.08	0.02 ± 0.01	38.06 ± 7.36	-0.11 ± 0.12	-4.07 ± 0.7	0.07 ± 0.2
605	4.71 ± 7.98	-44.63 ± 13.28	-1.86 ± 1.04	-16.37 ± 4.44	2.65 ± 0.64	-3.61 ± 1.21	1.92 ± 0.71	0.01 ± 0.01	-7.36 ± 10.24	0.16 ± 0.28	4.88 ± 2.48	0.26 ± 0.23
607	-1.57 ± 8.31	12.93 ± 37.11	3.4 ± 1.74	0.77 ± 4.96	0.36 ± 1.1	-6.75 ± 1.78	0.68 ± 4.55	0.01 ± 0.01	16.1 ± 11.46	2.88 ± 0.87	8.59 ± 4.17	0.5 ± 0.3
609	20.8 ± 6.35	-10.79 ± 20.45	-1.25 ± 1.25	-2.81 ± 6.26	-0.04 ± 0.85	-3.14 ± 1.17	-0.86 ± 2.4	0.03 ± 0.01	26.23 ± 7.46	0.03 ± 0.37	1.7 ± 3.44	-0.15 ± 0.31
630	-5.56 ± 8.69	-56.6 ± 32.69	1.39 ± 1.08	12.42 ± 3.87	10.1 ± 1.23	-3.43 ± 1.42	0.78 ± 1.1	0 ± 0.01	-1.28 ± 10.83	0.38 ± 0.16	-3.55 ± 1.41	0.77 ± 0.28
631	1.95 ± 3.53	-1.59 ± 17.54	2.24 ± 0.62	8.96 ± 3.11	1.72 ± 0.55	-2.97 ± 0.55	-4.28 ± 2.34	-0.01 ± 0.01	-1.15 ± 4.8	0.36 ± 0.29	12.88 ± 1.99	-0.33 ± 0.18

[a]evaluated across 3x3 pixels at 30-m resolution, this is an interaction variable

[b]percent area evaluated within 270-m radius circular buffer

[c]normalized difference vegetation index

[d]an index of eastness
