# Supplementary material for: Habitat selection by Dall’s sheep is influenced by multiple factors including direct and indirect climate effects
Source: PLoS One. 2021 Mar 18;16(3):e0248763. doi: 10.1371/journal.pone.0248763 (PMC7971871; doi:10.1371/journal.pone.0248763)
Supplement: S4 Table — (PDF) [file pone.0248763.s005.pdf]

S4 Table. Parameter estimates and standard errors (SE) for each habitat and climate variable in the best supported model for spring by individual Dall's sheep females (*Ovis dalli dalli*). Values are shown for individuals in the North and South regions within Lake Clark National Park and Preserve, Alaska during 2006-2007. See Table 3 in the main text for mean values of parameter estimates and standard errors of habitat variables. See S1 Table for description of habitat variables.

Individual	elevation ± SE	distance to escape terrain ± SE	ruggedness[a] ± SE	slope ± SE	alpine dwarf shrub[b] ± SE	shrub/scrub[b] ± SE	snow depth ± SE	solar radiation index ± SE	air temperature ± SE	wind speed ± SE	NDVI[c] ± SE	snow extent[b] ± SE
North												
613	-1.47 ± 5	-0.8 ± 0.06	-0.46 ± 1.5	5.32 ± 0.71	3.49 ± 0.41	-0.39 ± 0.65	-0.28 ± 1.37	0.33 ± 0.33	1.78 ± 4.97	-0.01 ± 0.1	-2.57 ± 0.93	0.15 ± 0.46
615	48.3 ± 6.6	-79.4 ± 20.8	3.16 ± 1.31	2.27 ± 1.01	0.75 ± 0.32	0.16 ± 0.5	-0.82 ± 1.36	1.53 ± 0.43	48.6 ± 6.24	-0.09 ± 0.08	-0.62 ± 0.62	-0.1 ± 0.42
616	20.12 ± 9.85	-51.17 ± 23.38	5 ± 1.45	1.47 ± 1.2	2.41 ± 0.41	-0.49 ± 0.83	-3.14 ± 1.52	0.62 ± 0.43	16.41 ± 9.63	-0.19 ± 0.1	0.8 ± 0.8	0.54 ± 0.48
619	4.24 ± 6.91	-45.08 ± 11.4	-3.24 ± 1.31	-1.77 ± 0.94	-0.41 ± 0.47	-3.36 ± 1.43	1.15 ± 1.19	0.82 ± 0.32	-6.81 ± 6.04	-0.16 ± 0.1	3.62 ± 0.95	-0.24 ± 0.46
620	40.96 ± 14.14	-62.12 ± 22.31	0.37 ± 2.13	-0.49 ± 1.16	-0.34 ± 0.5	-3.84 ± 0.94	-1.82 ± 2.31	-0.07 ± 0.56	34.64 ± 14.63	0.17 ± 0.12	2.66 ± 0.88	0.68 ± 0.61
622	9.02 ± 8.59	-48.61 ± 12.86	3.6 ± 1.2	2.65 ± 0.99	1.32 ± 0.34	-3.32 ± 0.99	-7.35 ± 1.62	2.32 ± 0.51	12.81 ± 8.01	0.09 ± 0.11	1.46 ± 0.81	1.74 ± 0.45
623	7.52 ± 15.54	-26.2 ± 11.59	0.92 ± 1.87	1.03 ± 1.1	0.76 ± 0.73	-1.94 ± 1.31	-2.05 ± 1.71	0.04 ± 0.41	3.64 ± 16.32	-0.14 ± 0.12	1.15 ± 0.86	-0.65 ± 0.52
624	6.23 ± 7.44	-65.55 ± 22.87	-0.59 ± 1.05	0.27 ± 1.15	2.96 ± 0.42	-7.7 ± 2.21	-1.28 ± 1.07	1.2 ± 0.33	2.59 ± 6.36	0.42 ± 0.14	2.41 ± 1.01	1.35 ± 0.48
626	-0.82 ± 7.22	-33 ± 12.23	-1.39 ± 1.07	1.67 ± 0.93	1.32 ± 0.32	0.35 ± 0.66	-2.83 ± 1.15	1.32 ± 0.35	-8.53 ± 6.93	-0.2 ± 0.07	-0.3 ± 0.72	0.68 ± 0.38
627	-5.28 ± 13.93	-41.14 ± 23.63	-5.13 ± 1.7	3.15 ± 1.51	-1.65 ± 0.66	-10.7 ± 3.42	-2.24 ± 1.98	1.73 ± 0.59	-1.04 ± 14.19	0.63 ± 0.2	4.07 ± 1.03	-1.19 ± 0.59
613	-6.59 ± 5.4	-3.41 ± 19.34	0.06 ± 1.52	5.47 ± 1.08	2.99 ± 0.43	-0.6 ± 0.63	-1.6 ± 2.86	0.83 ± 0.42	0.03 ± 5.41	0.76 ± 0.16	-5.83 ± 0.98	-1.3 ± 0.54
615	-4.58 ± 6.62	-42.82 ± 19.93	0.77 ± 1.36	2.52 ± 1.01	1.26 ± 0.36	-0.31 ± 0.59	-0.8 ± 2.97	1.49 ± 0.47	-6.49 ± 6.15	-0.2 ± 0.09	-0.66 ± 0.71	-1.7 ± 0.51
616	-12.54 ± 8.99	-70.34 ± 21.86	4.75 ± 1.22	2.02 ± 1.13	2.01 ± 0.34	-1.07 ± 0.65	-5.04 ± 3	0.07 ± 0.3	-13.67 ± 8.81	0.01 ± 0.1	0.96 ± 0.77	-2.08 ± 0.56
620	-1.46 ± 6.56	-15.27 ± 7.08	2.79 ± 1.29	4.99 ± 0.85	2.39 ± 0.35	-3.58 ± 0.94	-8.64 ± 5.07	0.06 ± 0.32	1.64 ± 6.11	-0.09 ± 0.1	-2.52 ± 0.88	-3.2 ± 0.7
622	-17.2 ± 6.09	-30.04 ± 9.79	-5.45 ± 1.32	0.08 ± 0.93	2.38 ± 0.38	-0.38 ± 0.98	-2.97 ± 2.15	0.46 ± 0.32	-28.73 ± 5.87	-0.17 ± 0.11	1.36 ± 0.88	0.59 ± 0.46
623	-0.78 ± 11.58	-22.63 ± 9.29	0.63 ± 1.59	3.54 ± 1.05	1.31 ± 0.44	-0.71 ± 0.85	-5.89 ± 4.47	0.38 ± 0.36	0.23 ± 10.91	0.65 ± 0.15	2.82 ± 0.83	-1.71 ± 0.63
624	29.19 ± 11.1	-79.05 ± 23.83	-2.19 ± 1.26	0.48 ± 1.26	0.96 ± 0.43	-9.95 ± 2.15	-7.71 ± 2.71	2.49 ± 0.49	31.89 ± 10.73	-0.02 ± 0.12	0.14 ± 0.95	-0.2 ± 0.53
626	-31.19 ± 10.86	-82.84 ± 16.41	-3.71 ± 1.18	0.11 ± 1.05	-0.21 ± 0.37	-0.49 ± 0.77	-1.95 ± 2.13	1.43 ± 0.39	-32.74 ± 10.47	-0.04 ± 0.09	0.41 ± 0.8	-1.18 ± 0.44
627	0.98 ± 14.28	-93.95 ± 21.64	-8.61 ± 1.64	-0.15 ± 1.55	2.78 ± 0.65	3.54 ± 1.32	-0.9 ± 2.9	1.06 ± 0.58	-22.9 ± 14.49	-0.05 ± 0.18	-0.28 ± 1.1	-1.37 ± 0.71
South												
501	6.21 ± 11.92	-21.43 ± 14.86	-1.12 ± 1.57	3.05 ± 1.12	-0.51 ± 0.56	1.24 ± 0.89	1.44 ± 2.55	1.35 ± 0.5	-6.47 ± 13.63	-0.01 ± 0.09	2.06 ± 1.07	-1.36 ± 0.69
604	5.16 ± 5.71	-40.1 ± 13.74	0.67 ± 0.9	2.16 ± 0.94	2.94 ± 0.5	-3.38 ± 0.8	0.43 ± 0.87	0.82 ± 0.36	7.76 ± 5.64	0.17 ± 0.13	-0.16 ± 0.99	0.99 ± 0.48
605	14.82 ± 8.25	-44.36 ± 14.27	7.32 ± 0.9	0.88 ± 0.97	0.34 ± 0.56	1.19 ± 0.73	2.57 ± 0.7	1.04 ± 0.36	14.05 ± 9.24	-0.05 ± 0.09	-0.15 ± 1.04	0.35 ± 0.54
607	17.17 ± 11.75	-25.86 ± 28.64	0.51 ± 1.45	2.12 ± 1.57	0.56 ± 0.75	-2.23 ± 1	-3.35 ± 2.6	2.03 ± 1.08	23.71 ± 12.57	0.07 ± 0.22	-1.42 ± 1.63	1.61 ± 0.76
609	-2.84 ± 9.34	-1.45 ± 14.27	2.43 ± 1.08	5.7 ± 1.2	0.41 ± 0.79	-3.19 ± 1.2	-2.92 ± 2.03	2.67 ± 0.76	2.86 ± 10.37	-0.05 ± 0.17	-1.56 ± 1.8	2.15 ± 0.7
630	38.82 ± 8.68	-8.05 ± 13.71	-0.35 ± 0.75	1.72 ± 1.11	0.58 ± 0.49	-3.41 ± 1.24	-6.07 ± 1.76	0.9 ± 0.4	29.25 ± 9.47	0.16 ± 0.11	1.05 ± 0.93	-0.76 ± 0.53
631	-5.01 ± 6.73	18.93 ± 13.61	5.13 ± 0.84	4.42 ± 0.79	0.69 ± 0.57	-3.92 ± 0.68	-1.13 ± 1.14	1.56 ± 0.53	-2.41 ± 7.66	-0.11 ± 0.12	-1.84 ± 0.88	3.03 ± 0.49
501	28.53 ± 11.98	-34.29 ± 17.53	-1.16 ± 1.42	0.53 ± 1.19	1.41 ± 0.54	0.68 ± 1.09	-5.6 ± 4.2	0.05 ± 0.39	17.13 ± 13.35	-0.17 ± 0.11	3.27 ± 1.21	-0.99 ± 0.88
605	-13.06 ± 4.44	-43.05 ± 11.57	4.75 ± 0.84	-0.28 ± 0.9	0.2 ± 0.5	-3.48 ± 1.12	1.05 ± 0.84	0.9 ± 0.3	-11.3 ± 4.71	0.45 ± 0.15	1.24 ± 0.81	-0.38 ± 0.52
607	-4.05 ± 11.27	-19.83 ± 31.02	2.13 ± 1.36	2.72 ± 1.55	-2.43 ± 0.92	-3.92 ± 1.17	-2.41 ± 2.89	1.78 ± 0.9	4.83 ± 12.2	0.72 ± 0.32	-1.29 ± 1.78	-1.33 ± 0.83
609	17.01 ± 10.34	-59.73 ± 27.03	-0.07 ± 1.39	2.66 ± 1.56	1.73 ± 0.72	-4.35 ± 1.36	-11.4 ± 3.13	2.87 ± 0.94	16.92 ± 11.26	-0.55 ± 0.23	-1.75 ± 1.61	-0.79 ± 0.77
630	20.43 ± 7.8	-7.96 ± 11.9	-0.26 ± 0.8	1.95 ± 1.08	1.75 ± 0.46	-3.26 ± 0.95	-1.69 ± 1.93	-0.73 ± 0.28	15.37 ± 8.1	0.73 ± 0.16	1.2 ± 0.85	-1.3 ± 0.6
631	22.3 ± 6.24	-15.04 ± 9.1	2.35 ± 0.65	6.3 ± 0.74	0.31 ± 0.4	-1.9 ± 0.48	-0.24 ± 1.07	2.37 ± 0.37	28.88 ± 6.66	-0.16 ± 0.1	-2.48 ± 0.7	-1.04 ± 0.42
701	18.4 ± 9.73	-26.93 ± 12.21	4.88 ± 1.08	0.57 ± 1.13	0.67 ± 0.46	-3.33 ± 1.09	-0.42 ± 1.82	0.4 ± 0.38	12.78 ± 10.86	-0.18 ± 0.09	-2.76 ± 1.11	-0.49 ± 0.74
702	-6.07 ± 23.96	-82.87 ± 26.79	-4.23 ± 2.82	-0.56 ± 2.45	1.75 ± 0.98	-12.8 ± 3.97	-2.4 ± 2.51	0.2 ± 0.61	-12.13 ± 26.28	-0.66 ± 0.15	10.24 ± 3.71	1.89 ± 1.16
705	33.37 ± 6.36	-21.63 ± 10.68	0.76 ± 0.7	5.95 ± 0.79	0.41 ± 0.4	-2.13 ± 0.56	-0.5 ± 1.05	2.83 ± 0.42	39.64 ± 6.71	-0.17 ± 0.11	-0.99 ± 0.75	0.36 ± 0.41

[a]evaluated across 7x7 pixels at 30-m resolution based on Sappington et al. (2007)

[b]percent area evaluated within 270-m radius circular buffer

[c]normalized difference vegetation index
