# Supplementary material for: Habitat selection by Dall’s sheep is influenced by multiple factors including direct and indirect climate effects
Source: PLoS One. 2021 Mar 18;16(3):e0248763. doi: 10.1371/journal.pone.0248763 (PMC7971871; doi:10.1371/journal.pone.0248763)
Supplement: S5 Table — (PDF) [file pone.0248763.s006.pdf]

S5 table. Parameter estimates and standard errors (SE) for each habitat and climate variable in the best supported model for summer by individual Dall's sheep females (*Ovis dalli dalli*). Values are shown for individuals in the North and South regions within Lake Clark National Park and Preserve, Alaska during 2006-2007. See Table 3 in the main text for mean values of parameter estimates and standard errors of habitat variables. See S1 Table for description of habitat variables.

Individual	elevation ± SE	distance to escape terrain ± SE	slope ± SE	mean slope x ruggedness[a] ± SE	alpine dwarf shrub[b] ± SE	shrub/scrub[b] ± SE	NDVI[c] ± SE
North							
613	6.42 ± 1.61	-62.64 ± 20.89	-0.44 ± 1.03	1.8 ± 1.56	2.23 ± 0.36	-2.91 ± 1.4	-1.27 ± 0.64
615	7.92 ± 1.5	-47.22 ± 16.76	0.1 ± 0.79	-0.35 ± 0.98	0.37 ± 0.34	-6.7 ± 1.95	1.69 ± 0.52
616	5.36 ± 1.36	-59.41 ± 17.98	-1.57 ± 0.85	3.11 ± 1.12	1.08 ± 0.39	-8.02 ± 2.5	1.8 ± 0.59
619	1.74 ± 1	-45.18 ± 10.54	-1.29 ± 0.72	0.18 ± 1.04	-0.82 ± 0.45	-2.61 ± 1.21	-1.97 ± 0.63
620	-4.45 ± 1.28	-51.07 ± 16.59	0.01 ± 0.9	-1.77 ± 1.24	1.31 ± 0.32	-12.14 ± 2.48	-1.2 ± 0.6
622	-3.68 ± 1	-45.19 ± 10.62	-0.29 ± 0.71	0.35 ± 1.1	0.06 ± 0.34	-7.4 ± 1.84	-1.65 ± 0.56
623	3.91 ± 1.98	-27.37 ± 7.63	-1.42 ± 0.75	-1.37 ± 2.21	0.91 ± 0.42	0.08 ± 0.89	-0.34 ± 0.55
624	5.05 ± 1.09	-34.24 ± 10.11	-1.11 ± 0.73	1.46 ± 0.97	2.42 ± 0.29	-0.5 ± 0.76	0.73 ± 0.46
626	10.3 ± 1.31	-8.22 ± 7.08	1.03 ± 0.63	-3.31 ± 1.24	2.11 ± 0.3	1.99 ± 0.53	1.08 ± 0.42
627	14.58 ± 1.91	-44.95 ± 12.97	-0.4 ± 0.88	-7.13 ± 1.63	0.53 ± 0.41	-0.46 ± 0.73	0.56 ± 0.52
613	12.76 ± 1.75	-27.07 ± 18.43	0.91 ± 0.97	4.57 ± 1.62	1.96 ± 0.39	3.69 ± 0.67	1.16 ± 0.44
615	2.32 ± 1.34	-127.14 ± 18.6	-3.39 ± 0.71	2.72 ± 0.89	-1.08 ± 0.44	-5.39 ± 1.73	1.83 ± 0.32
616	-3.81 ± 1.25	-71.15 ± 18.27	-1.03 ± 0.85	4.56 ± 0.99	-1.26 ± 0.34	-8.66 ± 1.71	1.83 ± 0.35
620	-6.1 ± 1.22	-7.41 ± 6.22	1.06 ± 0.63	4.63 ± 0.79	1.21 ± 0.32	-9.45 ± 1.74	0.26 ± 0.35
622	2.11 ± 0.97	-47.97 ± 11.57	-0.47 ± 0.74	-2.29 ± 1.15	0.12 ± 0.39	-5.11 ± 1.68	-0.5 ± 0.4
623	-1.42 ± 2.03	-4.04 ± 11.2	1.11 ± 0.79	8.11 ± 1.49	-1.3 ± 0.43	-2.48 ± 0.99	0.48 ± 0.44
624	8.8 ± 1.28	-13.47 ± 11.67	1.13 ± 0.74	-2.3 ± 0.97	1.26 ± 0.39	-1.43 ± 1.23	-0.71 ± 0.43
626	7.5 ± 1.27	-19.56 ± 6.81	-0.46 ± 0.6	-1.71 ± 1.2	1.77 ± 0.3	1.42 ± 0.56	1.45 ± 0.31
627	12.22 ± 1.97	-52.43 ± 12.79	-0.92 ± 0.89	-6.11 ± 1.65	1.18 ± 0.45	3.96 ± 0.5	0.84 ± 0.33
South							
501	13.8 ± 1.78	-11.22 ± 11.47	1.18 ± 0.86	5.78 ± 4.75	0.22 ± 0.43	-2.23 ± 1.1	0.02 ± 0.53
604	11.57 ± 1.4	0.82 ± 10.18	-0.18 ± 0.7	3.08 ± 1.64	-6.97 ± 2.69	-0.67 ± 2.08	0.76 ± 1.19
605	12.16 ± 1.4	-0.14 ± 6.11	-1.95 ± 0.65	-5.88 ± 3.17	-0.57 ± 0.45	-15.37 ± 8.6	-4.31 ± 1.15
607	1.13 ± 2.05	2.35 ± 16.53	0.55 ± 1.04	4.04 ± 2.54	-4.5 ± 1.86	-4.96 ± 3.83	4.66 ± 1.73
609	3.09 ± 1.56	-7.38 ± 12.84	0.39 ± 1.08	5.14 ± 3.56	0.87 ± 0.91	-2.43 ± 1.72	-1.87 ± 2.04
630	6.17 ± 1.8	-16.56 ± 13.68	0.5 ± 0.81	-2.52 ± 1.93	-0.09 ± 1.1	0.44 ± 2.16	2.27 ± 0.83
631	2.62 ± 0.82	-58.6 ± 9.69	-1.14 ± 0.62	1.83 ± 1.6	-2.33 ± 0.83	-5.67 ± 1.8	-2.33 ± 0.94
501	15.68 ± 1.92	-14.92 ± 12.56	1.61 ± 0.89	11.57 ± 4.67	0.27 ± 0.59	-0.51 ± 1.04	1.12 ± 0.46
605	7.57 ± 1.43	1.07 ± 7.28	-0.84 ± 0.64	9.48 ± 2.18	-2.4 ± 0.58	-7.19 ± 3.18	2.39 ± 0.48
607	-1.63 ± 2.07	-57.43 ± 25.45	-1.1 ± 1.19	7.76 ± 2.34	-6.03 ± 2	-20.71 ± 37.4	1.08 ± 1.4
609	-1.23 ± 1.32	27.65 ± 6.64	3.1 ± 0.89	17.93 ± 2.38	2.67 ± 0.88	-8.38 ± 4.24	-2.07 ± 0.89
630	6.28 ± 1.68	-8.51 ± 12.72	2.77 ± 0.89	0.56 ± 1.94	0.75 ± 0.71	-3.63 ± 1.64	1.5 ± 0.49
631	2.18 ± 0.8	-47.48 ± 7.88	0.43 ± 0.58	-0.65 ± 0.94	-1.28 ± 0.73	-4.8 ± 1.84	-0.73 ± 0.44
701	16.67 ± 1.72	6.1 ± 6.76	-1.45 ± 0.67	7.57 ± 2.89	-0.8 ± 0.42	-5.91 ± 1.5	0.67 ± 0.46
705	3.31 ± 0.81	-36.77 ± 7.1	0.6 ± 0.56	-0.57 ± 0.93	-2.15 ± 0.87	-3.24 ± 1.43	-0.72 ± 0.45

[a]evaluated across 3x3 pixels at 30-m resolution, this is an interaction variable

[b]percent area evaluated within 270-m radius circular buffer

[c]normalized difference vegetation index
