# Supplementary material for: Habitat selection by Dall’s sheep is influenced by multiple factors including direct and indirect climate effects
Source: PLoS One. 2021 Mar 18;16(3):e0248763. doi: 10.1371/journal.pone.0248763 (PMC7971871; doi:10.1371/journal.pone.0248763)
Supplement: S1 Text — (PDF) [file pone.0248763.s007.pdf]

S1 Text. Summary of SnowModel simulation

Aycrigg, J.L., A. G. Wells, E. O. Garton, B. Magipane, G. E. Liston, L. R. Prugh, and J. L. Rachlow. Habitat selection by Dall's sheep is influenced by direct and indirect climate effects.

Model description

SnowModel

Model simulations were performed using SnowModel (Liston and Elder 2006a, Liston et al. 2020), a spatially-distributed snow-evolution modeling system designed for application in all landscapes, climates, and conditions where snow occurs. It is an aggregation of four sub-models: EnBal (Liston 1995; Liston et al. 1999) calculates surface energy exchanges and snowmelt; SnowPack (Liston and Hall 1995; Liston and Mernild 2012) is a multi-layer snowpack sub-model that simulates snow depth and water-equivalent evolution; SnowTran-3D (Liston and Sturm 1998; Liston et al. 2007) accounts for snow redistribution by wind; and SnowAssim (Liston and Hiemstra 2008) is available to assimilate field and remote sensing datasets.

SnowModel is designed to run on grid increments of 1-m to 500-m and temporal increments of 10-minutes to 1-day. It can be applied using much larger grid increments (up to 10s of km) if the inherent loss in high-resolution (subgrid) information (Liston 2004) is acceptable. Processes simulated by SnowModel include snow precipitation; blowing-snow redistribution and sublimation; interception, unloading, and sublimation within forest canopies; snow-density evolution; and snowpack ripening and melt. SnowModel incorporates first-order physics required to simulate snow evolution within each of the global snow classes (i.e., Ice, Tundra, Taiga, Warm Forest [or Alpine], Prairie, Maritime, and Ephemeral) defined by Sturm et al. (1995) and G. E. Liston and M. Sturm (2016, unpublished manuscript). Required SnowModel

inputs include temporally-variant precipitation, wind speed and direction, air temperature, and relative humidity obtained from meteorological stations and/or an atmospheric model located within or near the simulation domain. Spatially-distributed, time-invariant topography and land cover are also necessary.

MicroMet

Meteorological forcings required by SnowModel are provided by MicroMet (Liston and Elder 2006b), a quasi-physically-based, high-resolution (e.g., 1-m to 10-km horizontal grid increment), meteorological distribution model. MicroMet is a data assimilation and interpolation model that utilizes meteorological station datasets and/or gridded atmospheric model or (re)analyses datasets. MicroMet minimally requires near-surface air temperature, relative humidity, wind speed and direction, and precipitation data. The model uses known relationships among meteorological variables and the surrounding landscape (primarily topography) to distribute those variables over any given landscape in physically plausible and computationally efficient ways (Liston and Elder 2006b). MicroMet performs two kinds of adjustments to the meteorological data; 1) all available data fields, at a given time, are spatially interpolated over the domain, and 2) physically based sub-models are applied to each MicroMet variable to quantify topographic, elevation, and vegetation effects at any given point in space and time. At each time step, MicroMet simulates and distributes air temperature, relative humidity, wind speed, wind direction, incoming solar radiation, incoming longwave radiation, surface pressure, and precipitation, and makes them accessible to SnowModel.

MicroMet and SnowModel constitute a physically-based modeling system that creates value-added snow information (e.g., snow depth, snow density, snow melt rate, snow thermal properties, snow cover duration, sublimation) from basic meteorological variables (e.g., air

temperature, humidity, precipitation, wind). The products yielded are based on our physical understanding of snow-evolution processes and features, and their interactions with the atmosphere and surrounding land surface. MicroMet and SnowModel have been used to distribute observed and modeled meteorological variables and evolve snow distributions over complex arctic and alpine terrains, such as Alaska, Arctic Canada, Siberia, Norway, Greenland, and Antarctica as part of a wide variety of terrestrial modeling studies (e.g., Liston and Sturm 1998, 2002; Greene et al. 1999; Liston et al. 2002, 2007, 2016; Hasholt et al. 2003; Bruland et al. 2004; Mernild et al. 2006, 2011; Liston and Hiemstra 2008; Mernild and Liston 2010; Suzuki et al. 2015b; Fletcher et al. 2012; Stuefer et al. 2013; Pedersen et al. 2015).

Model simulation

Model configuration and simulation domain

SnowModel simulations were performed for the period 1 September 2005 through 31 August 2008 (1096 days) over a spatial domain that covered an 88-km by 124-km area in southwest Alaska (Figure S1). Model simulations were performed using a 90-m horizontal grid increment over the domain (979 and 1383 grid cells in the x and y directions, respectively; or ~1.35 million grid cells). This 90-m grid increment strikes a balance between available computer resources and the need to accurately represent the driving snow-distribution processes found within the simulation domain. In addition, the simulations used a 1-day time increment to keep computational requirements within acceptable limits.

Topographical data (10 m horizontal resolution) for the domain were obtained from the United States Geological Survey (USGS), National Elevation Dataset and regridded to the 90-m simulation grid (Figure 1). Vegetation data were obtained from the USGS National Land Cover Data database and regridded to the 90-m grid and reclassified to match SnowModel's defined

vegetation classes (Figure 2; Liston and Elder 2006a).

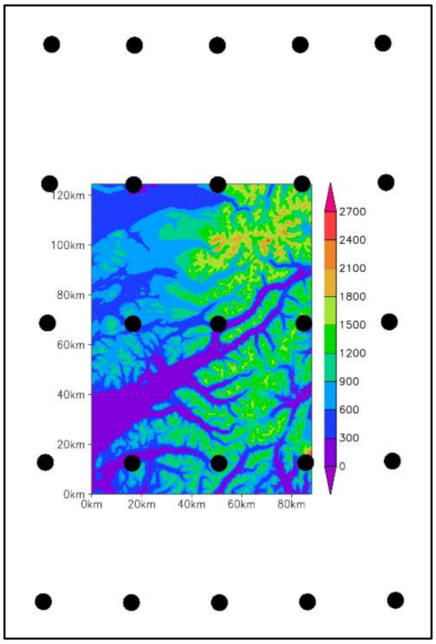

Figure 1. The Lake Clark National Park and Preserve (Alaska, USA) 88-km by 124-km simulation domain (color shades are topography; m), and MERRA atmospheric forcing locations (black dots).

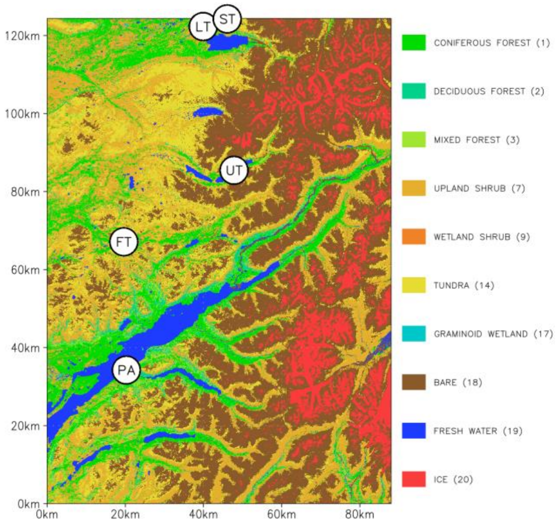

Figure 2. Land-cover distribution (see Liston and Elder 2006a for the available land-cover classes) for Lake Clark National Park and Preserve, Alaska, USA. The meteorological stations

(large circles with ID letters) used in the analyses as shown. The RAWS stations Port Alsworth (PA) and Stoney (ST) were used in the air temperature and wind speed analyses, and the SNOTEL stations PA and Lake Telaquana (LT) were used in the precipitation/snow depth analyses.

Meteorological forcing

Atmospheric forcing data were provided by NASA Modern Era Retrospective-Analysis for Research and Applications (MERRA) products (Bosilovich et al. 2008; Cullather and Bosilovich 2011; Rienecker et al. 2011; Lindsay et al. 2014). This reanalysis program has the specific goal of improving the representation of water cycle processes and features within the analyses while taking advantage of modern satellite era datasets. The latest version of the MERRA reanalysis (MERRA2) covers the period 1980-2016, on a 5/8° longitude by 1/2° latitude global grid. Hourly surface atmospheric forcing variables were available. The MERRA-2 reanalysis assimilates a wide range of satellite observations in addition to more conventional radiosonde, dropsonde, aircraft, and surface observations. Bosilovich et al. (2008) analyzed precipitation outputs from an early version of the MERRA reanalysis system and concluded the MERRA precipitation fields were an improvement over the previous generations of reanalysis.

In preparation for the model simulations, hourly, MERRA-2 10-m air temperature, specific humidity, and u and v wind components, and surface pressure and precipitation variables were aggregated to daily values. MERRA-2 grid points (n = 25, Figure 1) were used to force the MicroMet - SnowModel simulations. MicroMet then used these to create the daily, 90-m atmospheric forcing distributions required by SnowModel (air temperature, relative humidity, wind speed and direction, precipitation, and incoming solar and longwave radiation; see Liston and Elder (2006b) for additional details). Water-equivalent precipitation was provided from

MERRA-2, and MicroMet's temperature threshold parameterization was used to define whether rain or snow fell on each model grid cell. MicroMet ingested the MERRA-2 atmospheric variables and created the atmospheric forcing conditions on the 90-m SnowModel grid. The resulting 90-m atmospheric fields were ingested by SnowModel to simulate the daily time evolution and spatial distribution of water and energy fluxes and states. SnowModel-simulated variables included: surface (skin) temperature, albedo, outgoing longwave radiation, latent heat flux, sensible heat flux, liquid precipitation, solid precipitation, snowmelt, sublimation, snowmelt runoff, and snow water equivalent. In addition, we generated secondary products such as the timing and distribution of rain-on-snow events, changes in snow and growing season lengths, hydrologic budgets, and changes in surface energy exchanges.

As part of these simulations, SnowAssim was used to assimilate available SNOTEL snow depth data (Liston and Hiemstra 2008). This assimilation imposed a correction to the MERRA-2 water-equivalent precipitation inputs such that the SnowModel simulated snow water equivalent closely matched the SNOTEL observations. As part of the model integrations, the SNOTEL snow depths were converted to snow water equivalent using the Sturm et al. (2010) snow-classification snow-density sub-model. The resulting snow water equivalent was used to correct the water equivalent precipitation inputs. Then, because of a specific interest in snow depth, SnowModel’s snow water equivalent evolution was converted to snow depth evolution using the method of Sturm et al. (2010). The data assimilation was performed using the Port Alsworth and Lake Telaquana SNOTEL sites (PA and LT shown in Figure 2, respectively), using data available late in the snow accumulation season (Table 1).

Table 1: Late-winter SNOTEL snow-depth data used in the precipitation-correction assimilations for Lake Clark National Park and Preserve, Alaska, USA. The snow-water-equivalent values were obtained by applying the Sturm et al. (2010) taiga snow density (217 kg m^{-3}) to the SNOTEL snow-depth observations.

Station	Date	SNOTEL Snow Depth (cm)	SNOTEL Snow Water Equivalent (cm)	SnowModel Snow Depth (cm)	SnowModel Snow Water Equivalent (cm)
LT	4/5/2006	54.61	11.85	46.58	10.11
PA	4/7/2006	37.08	8.05	43.99	9.55
LT	4/2/2007	16.38	3.56	15.80	3.43
PA	4/3/2007	22.99	4.99	19.89	4.32
LT	3/13/2008	46.74	10.14	43.82	9.51
PA	3/14/2008	54.19	11.76	60.03	13.03

Results

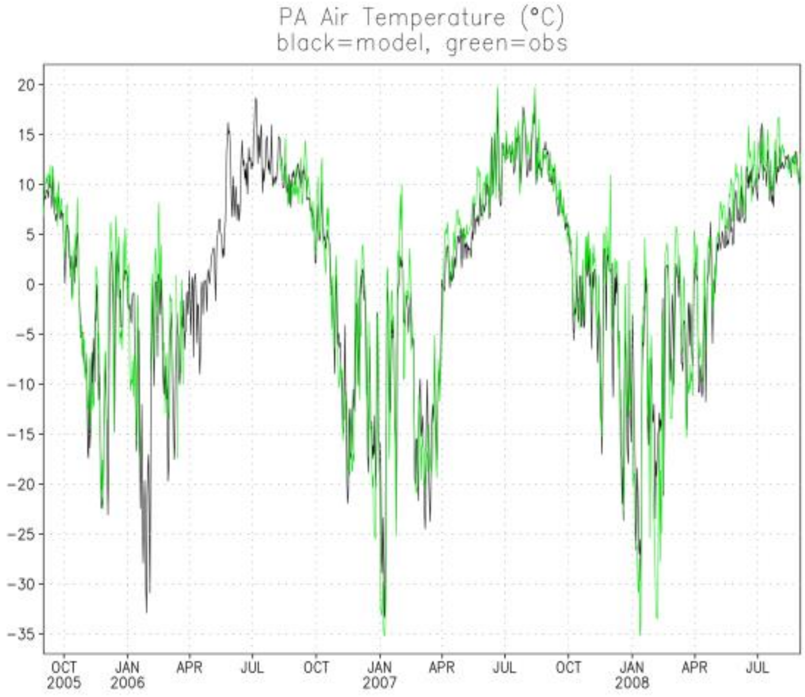

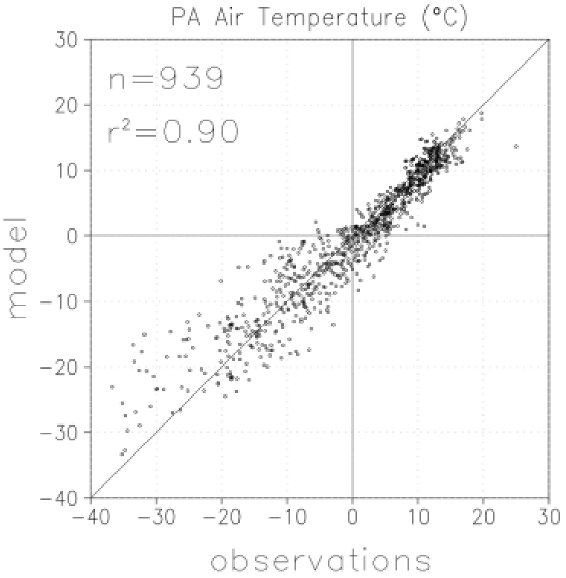

Figure 3. SnowModel for air temperature (degrees Celsius) results and correlation during October 2005 – August 2008 for Port Alsworth (PA).. Model results are shown in black and observed data are shown in green.

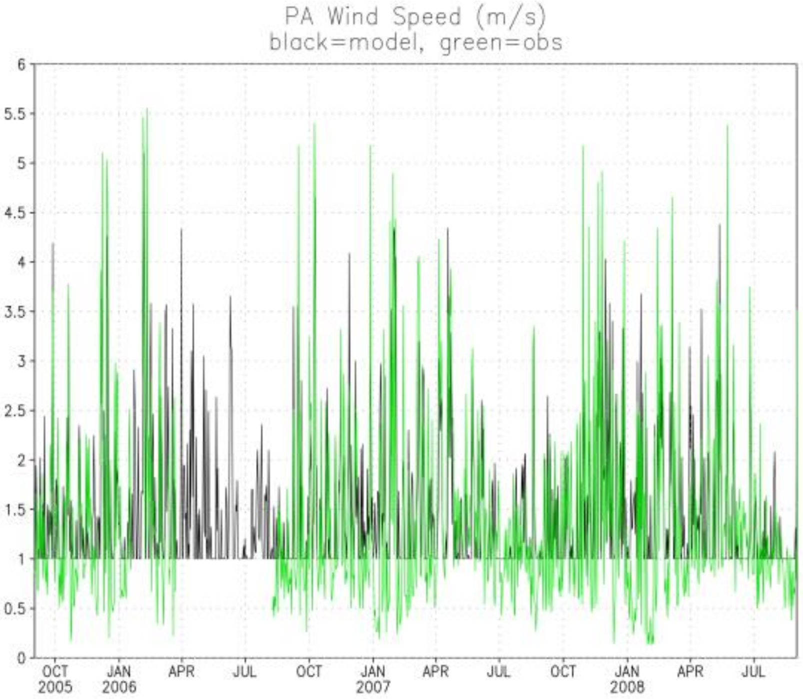

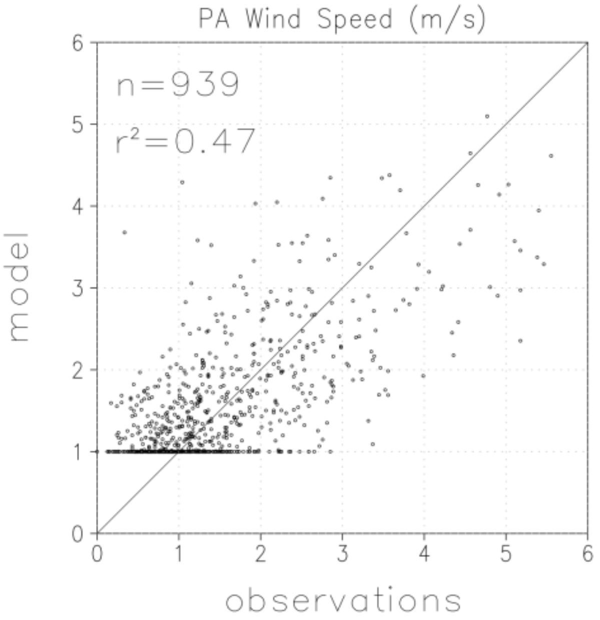

Figure 4. SnowModel for wind speed (meters/second) results and correlation during October 2005 – August 2008 for Port Alsworth (PA), Alaska. Model results are shown in black and observed data are shown in green. MicroMet does not allow wind speeds below 1 meter/second because low wind speeds violate some of the turbulent wind field assumptions in the model equations.

PA:

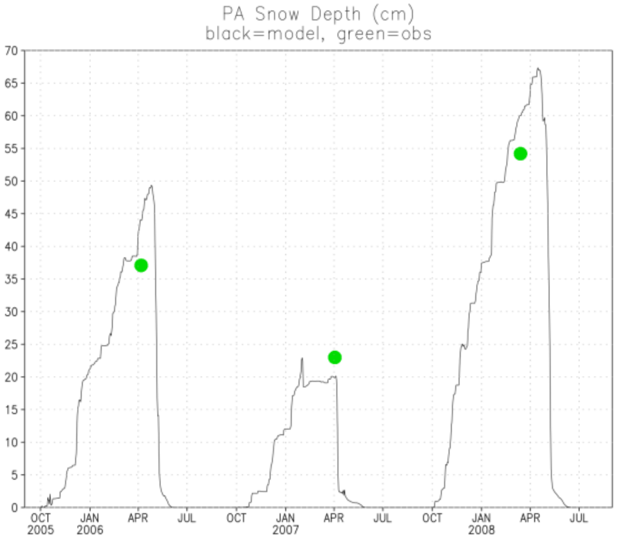

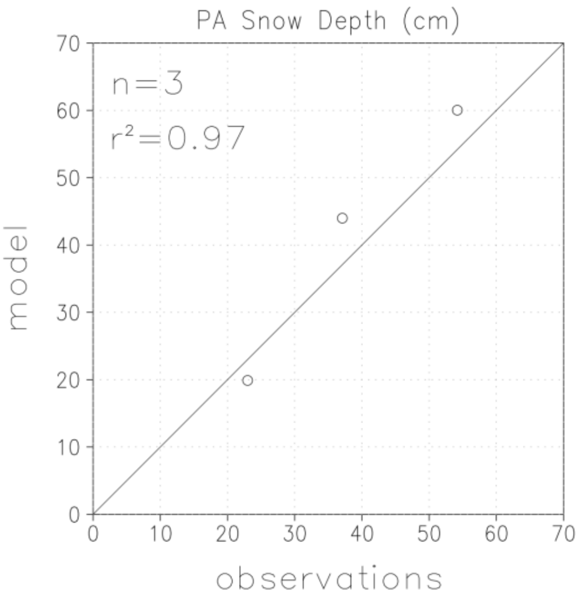
PA Snow Depth (cm)
n=3
r^2=0.97
model
observations
0
10
20
30
40
50
60
70

LT:

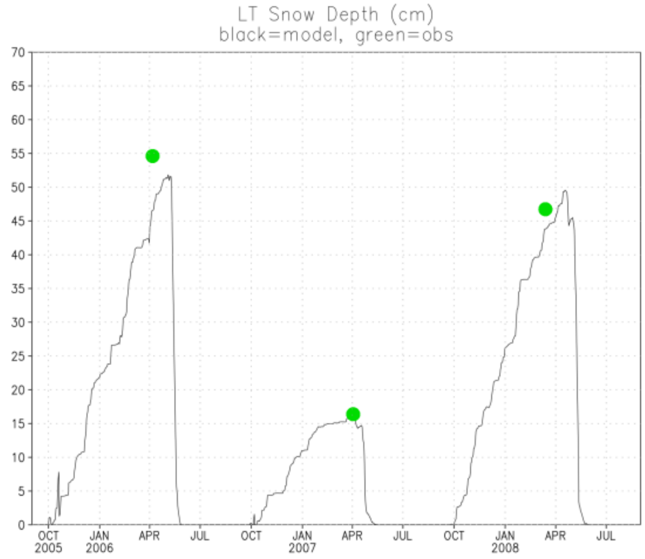
LT Snow Depth (cm)
black=model, green=obs
OCT 2005
JAN 2006
APR
JUL
OCT
JAN 2007
APR
JUL
OCT
JAN 2008
APR
JUL

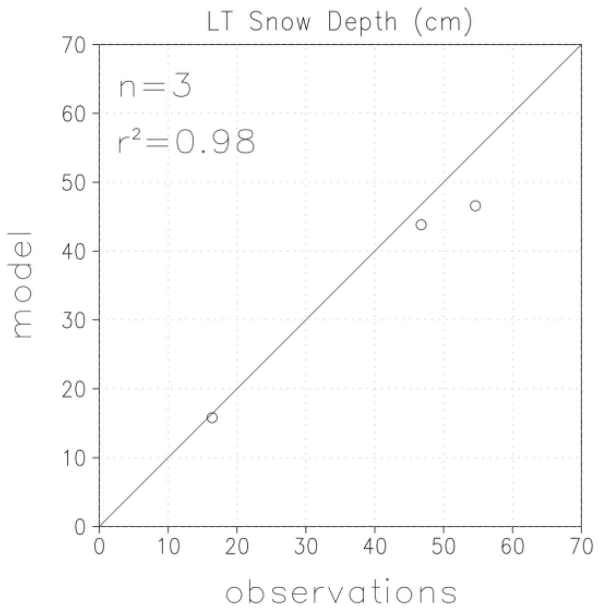

Figure 5. SnowModel for snow depth (centimeters) results and correlation during October 2005 – July 2008 for Port Alsworth (PA) and Lake Telaquana (LT), Alaska, USA. Model results are shown in black and observed data are shown in green. The snow water equivalent plots look similar to snow dept plots and only differ by the constant of the snow density. The data assimilation model pushes the simulations towards these snow depth observations. They are supposed to be close if the model is working as intended.

Landsat

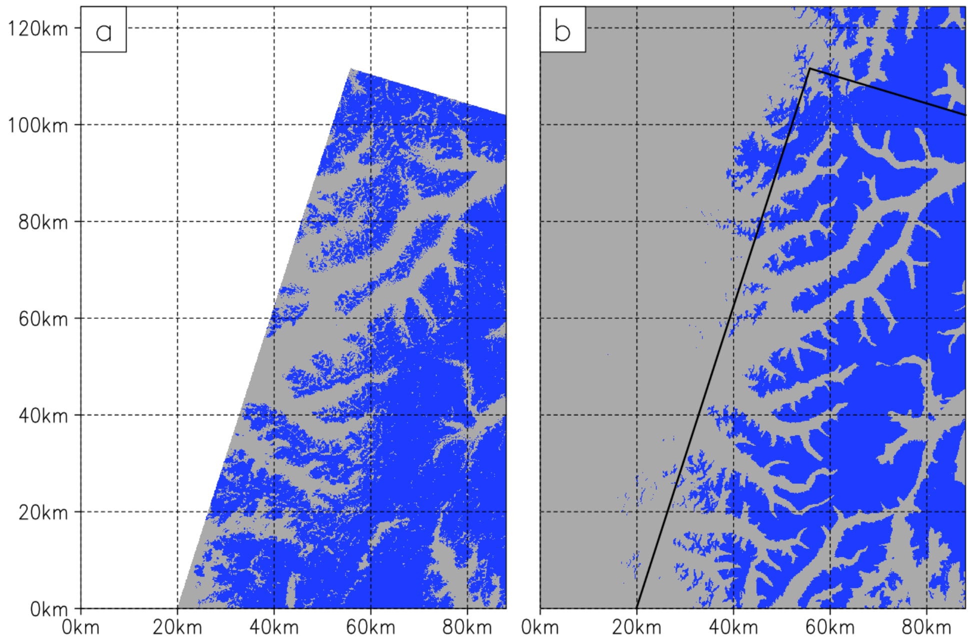

Figure 6. Landsat image LT50710182008170GLC00 from 18 June 2008 was used to compare the snow-covered area simulated by SnowModel on this same date. The Landsat image (a) is 37% snow-free and the SnowModel output (b) for the area coincident with the Landsat image indicates that 42% of the area is snow-free. The western half of the Landsat image had considerable cloud cover, so it was not used in the analysis. Grey is snow-free, blue is snow-covered.

Discussion

Overall, the model is representing the low-elevation snow evolution well. All of the available observational datasets match the model simulation very well (see figures above). However, we had no observations in the higher elevations in this domain, which means it is all under model control. We do not know the air temperature or the melt rates nor how the snowfall is different than in the lowlands. We also do not know how hard the wind blows at higher elevations and that has a direct influence on whether there are snow-free slopes open all winter because all of

the snow blows away in the wind on the windward slopes. This could make a difference in our high-elevation animal application. More data of any kind of meteorological or snow-related information high on the bare ridges could be valuable information in this study and improve the model output.

Model configurations specific to LCNPP

1. A wind increase with elevation was implemented in SnowModel. This was required in order to have blowing snow on the highest ridges (when driven with the relatively low-elevation MERRA-2 wind speeds). The presence of glaciers on lee slopes indicated this was an important component of the system. The wind speed increase is represented the same way that precipitation increases with elevation in the modeling system but using different parameters. For LCNPP, a general wind speed increase of 25% per 1-km elevation gain was used.

2. A moist lapse rate was applied to the simulation (as opposed to the relatively dry value for places like Colorado). Without this snow never melts in the highest elevations. The melting-snow albedo used in the simulation was the SnowModel default.

3. As part of the data assimilation, the MERRA-2 precipitation forcing was decreased by approximately 2/3 in order to reproduce the SNOTEL observations.

4. The precipitation increase with elevation parameter in SnowModel was reduced to 1/10th of typical values used in SnowModel.

5. Only the values for snow depth for Port Alsworth and Lake Telaquana SNOTEL sites were used in the assimilation. Furthermore, only the late-winter snow depth values were used. We believe these values best represented the regional precipitation distributions.

6. In the assimilation, we assumed the snow depth observations at the SNOTEL sites represented an area of 5-km radius around the observation point.

Literature cited

Bosilovich, M. G., J. Chen, F. R. Robertson, and R. F. Adler, 2008: Evaluation of global precipitation reanalyses. J. Applied Meteorol. and Climatology, 47, 2279-2299.

Bruland, O., G. E. Liston, J. Vonk, and A. Killingtveit, 2004: Modelling the snow distribution at two High-Arctic sites at Svalbard, Norway, and at a Sub-Arctic site in Central Norway. Nordic Hydrology, 35, 191-208.

Cullather, R. I., and M. Bosilovich, 2011: The moisture budget of the polar atmosphere in MERRA. J. Climate, 24, 2861-2879.

Fletcher, S. J., G. E. Liston, C. A. Hiemstra, and S. D. Miller, 2012: Assimilating MODIS and AMSR-E snow observations in a snow evolution model. Journal of Hydrometeorology, 13, 1475-1492.

Greene, E. M., G. E. Liston, and R. A. Pielke Sr., 1999: Simulation of above treeline snowdrift formation using a numerical snow-transport model. Cold Regions Sci. Tech., 30. 135-144.

Hoffman, M. J., A. G. Fountain, and G. E. Liston, 2016: Distributed modeling of ablation (1996-2011) and climate sensitivity on the glaciers of Taylor Valley, Antarctica. J. Glaciol., 1-15, doi: 10.1017/jog.2015.2.

Lindsay, R., M. Wensnahan, A. Schweiger, J. Zhang, 2014: Evaluation of seven different atmospheric reanalysis products in the Arctic. J. Climate, 27, 2588-2606.

Liston, G. E., 1995: Local advection of momentum, heat, and moisture during the melt of patchy snow covers. J. Applied Meteorol., 34, 1705-1715.

Liston, G. E., 2004: Representing subgrid snow cover heterogeneities in regional and global models. J. Climate, 17, 1381-1397.

Liston, G. E., and K. Elder, 2006a: A distributed snow-evolution modeling system (SnowModel). J. Hydrometeorology, 7, 1259-1276.

Liston, G. E., and K. Elder, 2006b: A meteorological distribution system for high-resolution terrestrial modeling (MicroMet). J. Hydrometeorology, 7, 217-234.

Liston, G. E., and D. K. Hall, 1995: An energy balance model of lake ice evolution. J. Glaciol., 41, 373-382.

Liston, G. E., and C. A. Hiemstra, 2008: A simple data assimilation system for complex snow distributions (SnowAssim). J. Hydrometeorology, 9, 989-1004.

Liston, G. E., and C. A. Hiemstra, 2011a: The changing cryosphere: Pan-Arctic snow trends (1979-2009). J. Climate, 24, 5691-5712.

Liston, G. E., and C. A. Hiemstra, 2011b: Representing grass- and shrub-snow-atmosphere interactions in climate system models. J. Climate, 24, 2061–2079.

Liston, G. E., and M. Sturm, 1998: A snow-transport model for complex terrain. J. Glaciology, 44, 498-516.

Liston, G. E., and M. Sturm, 2002: Winter precipitation patterns in arctic Alaska determined from a blowing-snow model and snow-depth observations. J. Hydrometeor., 3, 646-659.

Liston, G. E., J. -G. Winther, O. Bruland, H. Elvehøy, and K. Sand, 1999: Below-surface ice melt on the coastal Antarctic ice sheet. J. Glaciol., 45, 273-285.

Liston, G. E., J. -G. Winther, O. Bruland, H. Elvehøy, K. Sand, and L. Karlöf, 2000: Snow and blue-ice distribution patterns on the coastal Antarctic ice sheet. Antarctic Science, 12, 69-79.

Liston, G. E., J. P. McFadden, M. Sturm, and R. A. Pielke, Sr., 2002: Modeled changes in arctic tundra snow, energy, and moisture fluxes due to increased shrubs. Global Change

Biology, 8, 17-32.

Liston, G. E., R. B. Haehnel, M. Sturm, C. A. Hiemstra, S. Berezovskaya, and R. D. Tabler, 2007: Simulating complex snow distributions in windy environments using SnowTran-3D. Journal of Glaciology, 53, 241-256.

Liston, G. E., C. A. Hiemstra, K. Elder, and D. W. Cline, 2008: Meso-cell study area (MSA) snow distributions for the Cold Land Processes Experiment (CLPX). J. Hydrometeorology, 9, 957-976.

Liston, G. E., C. J. Perham, R. T. Shideler, and A. N. Cheuvront, 2016: Modeling snowdrift habitat for polar bear dens. Ecological Modelling, 320, 114-134.

Liston, G. E., P. Itkin, J. Stroeve, M. Tschudi, J. S. Stewart, S. H. Pedersen, A. K. Reinking, and K. Elder, 2020: A Lagrangian snow-evolution system for sea-ice applications (SnowModel-LG): Part I – model description. Journal of Geophysical Research – Oceans, in press.

Mernild, S. H., and G. E. Liston, 2010: The influence of air temperature inversions on snowmelt and glacier mass-balance simulations, Ammassalik Island, SE Greenland. J. Applied Meteorology and Climatology, 49, 47-67.

Mernild, S. H., G. E. Liston, B. Hasholt, and N. T. Knudsen, 2006: Snow-distribution and melt modeling for Mittivakkat Glacier, Ammassalik Island, Southeast Greenland. J. Hydrometeorology, 7, 808-824.

Mernild, S., H., B. Hasholt, and G. E. Liston, 2008: Climatic control on river discharge simulations, Zackenberg River drainage basin, northeast Greenland. Hydrological Processes, 22, 1932-1948.

Mernild, S. H., G. E. Liston, C. A. Hiemstra, K. Steffen, E. Hanna, and J. H. Christensen, 2009:

Greenland Ice Sheet surface mass-balance modelling and freshwater flux for 2007, and in a 1995–2007 perspective. Hydrological Processes, DOI: 10.1002/hyp.7354.

Mernild, S. H., G. E. Liston, C. A. Hiemstra, and J. H. Christensen, 2010: Greenland Ice Sheet surface mass-balance modeling in a 131-year perspective, 1950-2080. J. Hydrometeorology, 11, 3-25, DOI:10.1175/2009JHM1140.1.

Mernild, S. H., T. L. Mote, and G. E. Liston, 2011: Greenland ice sheet surface melt extent and trends, 1960–2010. Journal of Glaciology, 57, 621-628.

Mernild, S. H., G. E. Liston, and C. A. Hiemstra, 2014: Northern Hemisphere glacier and ice cap surface mass balance and contribution to sea level rise. J. Climate, 27, 6051-6073.

Mernild, S. H., D. M. Holland, D. Holland, A. Rosing-Asvid, J. C. Yde, G. E. Liston, and K. Steffen, 2015: Freshwater flux and spatiotemporal simulated runoff variability into Ilulissat Icefjord, West Greenland, linked to salinity and temperature observations near tidewater glacier margins obtained using instrumented ringed seals. J. Physical Oceanography, 45, 1426-1445.

Pedersen, S. H., G. E. Liston, M. P. Tamstorf, A. Westergaard-Nielsen, and N. M. Schmidt, 2015: Quantifying episodic snowmelt events in Arctic ecosystems. Ecosystems, 18, 839-856, doi: 10.1007/s10021-015-9867-8.

Rienecker, M. M., M. J. Suarez, R. Gelaro, R. Todling, J. Bacmeister, E. Liu, M. G. Bosilovich, S. D. Schubert, L. Takacs, G. -K. Kim, S. Bloom, J. Chen, D. Collins, A. Conaty, A. da Silva, et al., 2011: MERRA: NASA's Modern-Era Retrospective Analysis for Research and Applications. J. Climate, 24, 3624-3648.

Stuefer, S., D. Kane, and G. E. Liston, 2013: In situ snow water equivalent observations in the U.S. Arctic. Hydrology Research, 44, 21-34.

Sturm, M., J. Holmgren, and G. E. Liston, 1995: A seasonal snow cover classification system for local to global applications. J. Climate, 8, 1261-1283.

Sturm, M., B. Taras, G. E. Liston, C. Derksen, T. Jonas, and J. Lea, 2010: Estimating snow water equivalent using snow depth data and climate classes. J. Hydrometeorology, 11, 1380-1394.

Suzuki, K., Y. Kodama, T. Nakai, G. E. Liston, K. Yamamoto, T. Ohata, Y. Ishii, A. Sumida, T. Hara, and T. Ohta, 2011: Impact of land-use changes on snow in a forested region with heavy snowfall in Hokkaido, Japan. Hydrological Sciences Journal, 56, 443-467.

Suzuki, K., G. E. Liston, and Y. Kodama, 2015a: Variations in winter surface net shortwave radiation caused by land-use change in northern Hokkaido, Japan. Journal of Forest Research, 20, 281-292.

Suzuki, K., G. E. Liston, and K. Matsuo, 2015b: Estimation of continental-basin-scale sublimation in the Lena River basin, Siberia. Advances in Meteorology, Vol. 2015, Article ID 286206, 14 pages, http://dx.doi.org/10.1155/2015/286206.
