# Supplementary material for: Habitat selection by Dall’s sheep is influenced by multiple factors including direct and indirect climate effects
Source: PLoS One. 2021 Mar 18;16(3):e0248763. doi: 10.1371/journal.pone.0248763 (PMC7971871; doi:10.1371/journal.pone.0248763)
Supplement: S2 Text — (PDF) [file pone.0248763.s008.pdf]

S2 Text: Methods for variable reduction and selection of seasonal models

Aycrigg, J.L., A. G. Wells, E. O. Garton, B. Magipane, G. E. Liston, L. R. Prugh, and J. L. Rachlow. Habitat selection by Dall's sheep is influenced by direct and indirect climate effects.

Modeling steps

We used the following steps to generate seasonal population-level models of habitat selection by female Dall's sheep in Lake Clark National Park and Preserve, Alaska, USA:

1. For each season (Winter, Spring and Summer), we first evaluated 4 Ecological Hypotheses (Nutrition, Security, Movement and Thermal; See Fig. 1 in main text for details) by creating a set of models using single habitat and climate variables, combinations of multiple variables, a global model that included all variables (excluding highly correlated ones), and a null model (see Table 1 in main text).

2. We ran a synoptic model for each individual and each combination of habitat variables for each Season x Ecological Hypothesis combination (Horne et al. 2008, n=10).

3. To evaluate population-level affects, we used an information theoretic approach to select the model that best fit the data by summing Akaike's information criterion corrected for small sample sizes values (AIC_c) for each model across individuals for each Season x Ecological Hypothesis combination (Horne et al. 2008). We calculated delta AIC_c to identify models with the greatest support within each Season x Ecological Hypothesis combination (Burnham and Anderson 2002).

4. For each season (Winter, Spring, and Summer), we next evaluated which combination of Ecological Hypotheses best explained habitat selection by creating model sets that included the best model for each single Ecological Hypothesis (identified in the preceding step), combinations of Ecological Hypotheses, a global model, and a null model (n=16 for Winter and Spring; n=4 for Summer).

5. We ran a synoptic model for each model set, which was a combination of the Ecological Hypotheses within each Season (Tables 1-3 below).

6. We used an information theoretic approach to select the model set that best fit the data by summing AIC_c values for each model set across individuals. We calculated values for delta AIC_c to identify the final models for each season with the greatest support (Tables 1-3 below).

We completed these 6 steps for each year for which complete data were available (2006 and 2007) and for sheep in each region (North and South of Lake Clark), and for both years and

regions combined. If habitat and climate variables for a best fit models differed between regions, we summed the AIC_c values from the synoptic model output across regions to determine which variable or combination of variables best fit the data overall (Burnham and Anderson 2002)

Complete model results are presented in S2 Table, and the final models are summarized in Table 2 in the main text.

Table 1. Ecological hypotheses evaluated to explain habitat selection during winter by female Dall's sheep (*Ovis dalli dalli*) in Lake Clark National Park and Preserve (LCNNP), Alaska, USA. Habitat and climate variables, number of parameters (K), and delta AIC_c values are reported for sheep in the North, South, and both regions of LCNPP combined (see Fig. 2 in main text). The global model, which best fit the data, is shown in bold. Models are ordered by ΔAIC_c values from lowest to highest by both regions. See S1 Table for details about each habitat variable.

**Ecological Hypotheses**	**Habitat and Climate Variables**	**K**	**North**	**South**	**Both**
			**ΔAIC_c**	**ΔAIC_c**	**ΔAIC_c**
**Security + Thermal + Movement + Nutrition**	**alpine dwarf scrub + shrub/scrub + NDVI + snow depth + elevation + slope + mean slope x ruggedness + distance to escape terrain + sine of aspect + solar radiation + wind speed + air temperature**	**12**	**0**	**0**	**0**
Security + Thermal + Nutrition	alpine dwarf scrub + shrub/scrub + NDVI + snow depth + elevation + slope + mean slope x ruggedness + distance to escape terrain + solar radiation + wind speed + air temperature	11	72	48	120
Security + Movement + Nutrition	alpine dwarf scrub + shrub/scrub + NDVI + snow depth + elevation + slope + mean slope x ruggedness + distance to escape terrain + sine aspect	9	111	75	185
Security + Nutrition	alpine dwarf scrub + shrub/scrub + NDVI + snow depth + elevation + slope + mean slope x ruggedness + distance to escape terrain	8	220	84	304
Thermal + Movement + Nutrition	alpine dwarf scrub + shrub/scrub + NDVI + snow depth + sine aspect + solar radiation + wind speed + air temperature	8	356	188	544
Thermal + Nutrition	alpine dwarf scrub + shrub/scrub + NDVI + snow depth + solar radiation + wind speed + air temperature	7	443	192	635
Security + Thermal + Movement	elevation + slope + mean slope x ruggedness + distance to escape terrain + snow depth + sine aspect + solar radiation + wind speed + air temperature	9	390	338	729

Security + Thermal	elevation + slope + mean slope x ruggedness + distance to escape terrain + solar radiation + wind speed + snow depth + air temperature	8	429	352	781
Security	elevation + slope + (mean slope x ruggedness + distance to escape terrain	4	451	378	830
Security + Movement	elevation + slope + (mean slope x ruggedness + distance to escape terrain + snow depth + sine aspect	6	494	409	902
Nutrition	alpine dwarf scrub + shrub/scrub + NDVI + snow depth	4	745	248	994
Movement + Nutrition	alpine dwarf scrub + shrub/scrub + NDVI + snow depth + sine aspect	5	849	254	1103
Thermal	solar radiation + wind speed + snow depth + air temperature	4	744	470	1214
Thermal + Movement	snow depth + sine aspect + solar radiation + wind speed + air temperature	5	812	588	1400
Movement	snow depth + sine aspect	2	1339	781	2120
Null		0	1392	841	2233

Table 2. Ecological hypotheses evaluated to explain habitat selection during spring by female Dall's sheep (*Ovis dalli dalli*) in Lake Clark National Park and Preserve (LCNNP), Alaska, USA. Habitat and climate variables, number of parameters (K), and delta AIC_c values are reported for the North, South, and both regions of LCNPP combined (see Fig. 2 in main text). The global model, which best fit the data, is shown in bold. Models are ordered by ΔAIC_c values from lowest to highest by both regions. See S1 Table for details about each habitat variable.

**Ecological Hypotheses**	**Habitat and Climate Variables**	**K**	**North**	**South**	**Both**
			**ΔAICc**	**ΔAICc**	**ΔAIC_c**
**Security + Thermal + Movement + Nutrition**	**alpine dwarf scrub + shrub/scrub + NDVI + snow extent + elevation + distance to escape terrain + ruggedness + slope + snow depth + solar radiation + wind speed + air temperature**	**12**	**0**	**0**	**0**
Security + Thermal + Nutrition	alpine dwarf scrub + shrub/scrub + NDVI + snow extent + elevation + distance to escape terrain + ruggedness + slope + solar radiation + wind speed + air temperature + snow depth	12	29	19	48
Security + Movement + Nutrition	alpine dwarf scrub + shrub/scrub + NDVI + snow extent + elevation + distance to escape terrain + ruggedness + slope + snow depth	9	419	411	831
Security + Nutrition	elevation + distance to escape terrain + ruggedness + slope + alpine dwarf scrub + shrub/scrub + NDVI + snow extent	8	428	477	905

Security + Thermal	elevation + distance to escape terrain + ruggedness + slope + solar radiation + wind speed + snow depth + air temperature	8	800	375	1175
Security + Thermal + Movement	elevation + distance to escape terrain + ruggedness + slope + snow depth + snow extent + solar radiation + wind speed + air temperature	9	691	853	1544
Security + Movement	elevation + distance to escape terrain + ruggedness + slope + snow depth + snow extent	6	1154	723	1877
Thermal + Movement + Nutrition	alpine dwarf scrub + shrub/scrub + NDVI + snow extent + snow depth + solar radiation + wind speed + air temperature	8	1276	1091	2367
Thermal + Nutrition	alpine dwarf scrub + shrub/scrub + NDVI + snow extent + solar radiation + wind speed + snow depth + air temperature	8	1304	1133	2437
Security	elevation + distance to escape terrain + ruggedness + slope	4	1550	949	2500
Movement + Nutrition	alpine dwarf scrub + shrub/scrub + NDVI + snow extent + snow depth	5	1797	1773	3569
Nutrition	alpine dwarf scrub + shrub/scrub + NDVI + snow extent	4	1872	1853	3725
Thermal + Movement	snow depth + snow extent + solar radiation + wind speed + air temperature	5	2362	1386	3747
Thermal	solar radiation + wind speed + snow depth + air temperature	4	2525	1520	4045
Movement	snow depth + snow extent	2	3203	2403	5605
Null		0	3608	2834	6442

Table 3. Ecological models evaluated to explain habitat selection during summer by female Dall's sheep (*Ovis dalli dalli*) in Lake Clark National Park and Preserve (LCNPP), Alaska, USA. Habitat and climate variables, number of parameters (K), and delta AIC_c values are reported for the North, South, and both regions of LCNNP combined (see Fig. 2 in main text). The global model, which best fit the data, is shown in bold. Models are ordered by ΔAIC_c values from lowers to highest by both regions. See S1 Table for details about each habitat variable.

**Ecological Hypotheses**	**Habitat and Climate Variables**	**K**	**North**	**South**	**Both**
			**ΔAICc**	**ΔAICc**	**ΔAIC_c**
**Security + Nutrition**	**elevation + slope + mean slope x ruggedness + distance to escape terrian + alpine dwarf scrub + shrub/scrub + NDVI**	**7**	**0**	**0**	**0**
Security	elevation + slope + mean slope x ruggedness + distance to escape terrian	4	7345	255	7599
Nutrition	alpine dwarf scrub + shrub/scrub + NDVI	3	7762	1411	9173
Null		0	9177	2996	12173

Literature Cited

Burnham, K. P., and D. R. Anderson. 2002. Model Selection and Multimodel Inference: A Practical Information-Theoretic Apporach. Second edi. Springer.

Horne, J. S., E. O. Garton, and J. L. Rachlow. 2008. A synoptic model of animal space use: Simultaneous estimation of home range, habitat selection, and inter/intra-specific relationships. Ecological Modelling 214:338–348.
